# Polyclonal regeneration of mouse bone marrow endothelial cells after irradiative conditioning

**DOI:** 10.1016/j.celrep.2024.114779

**Published:** 2024-10-26

**Authors:** Izabella Skulimowska, Jan Morys, Justyna Sosniak, Monika Gonka, Gunsagar Gulati, Rahul Sinha, Kacper Kowalski, Sylwester Mosiolek, Irving L. Weissman, Alicja Jozkowicz, Agata Szade, Krzysztof Szade

**Affiliations:** 1 Department of Medical Biotechnology, Faculty of Biochemistry, Biophysics and Biotechnology, Jagiellonian University, Gronostajowa 7, 30-387 Krakow, Poland; 2 Laboratory of Stem Cell Biology, Faculty of Biochemistry, Biophysics and Biotechnology, Jagiellonian University, Gronostajowa 7, 30-387 Krakow, Poland; 3 Doctoral School of Exact and Natural Sciences, Faculty of Biochemistry, Biophysics and Biotechnology, Jagiellonian University, Gronostajowa 7, 30-387 Krakow, Poland; 4 Institute for Stem Cell Biology and Regenerative Medicine, Stanford University, Stanford, CA 94305, USA

**Keywords:** hematopoietic stem cells, bone marrow niche, transplantation, conditioning, sinusoids, bone marrow endothelial cells, bone marrow regeneration, irradiation

## Abstract

Bone marrow endothelial cells (BM-ECs) are the essential components of the BM niche and support the function of hematopoietic stem cells (HSCs). However, conditioning for HSC transplantation causes damage to the recipients’ BM-ECs and may lead to transplantation-related morbidity. Here, we investigated the cellular and clonal mechanisms of BM-EC regeneration after irradiative conditioning. Using single-cell RNA sequencing, imaging, and flow cytometry, we revealed how the heterogeneous pool of BM-ECs changes during regeneration from irradiation stress. Next, we developed a single-cell *in vitro* clonogenic assay and demonstrated that all EC fractions hold a high potential to reenter the cell cycle and form vessel-like structures. Finally, we used Rainbow mice and a machine-learning-based model to show that the regeneration of BM-ECs after irradiation is mostly polyclonal and driven by the broad fraction of BM-ECs; however, the cell output among clones varies at later stages of regeneration.

## Highlights

• Bone marrow endothelial cells (BM-ECs) have high potential to reenter cell cycle
• Regeneration of BM-ECs after irradiation is highly polyclonal
• scRNA-seq reveals dynamic remodeling of BM-ECs after irradiation

## Graphical abstract

**Figure:**
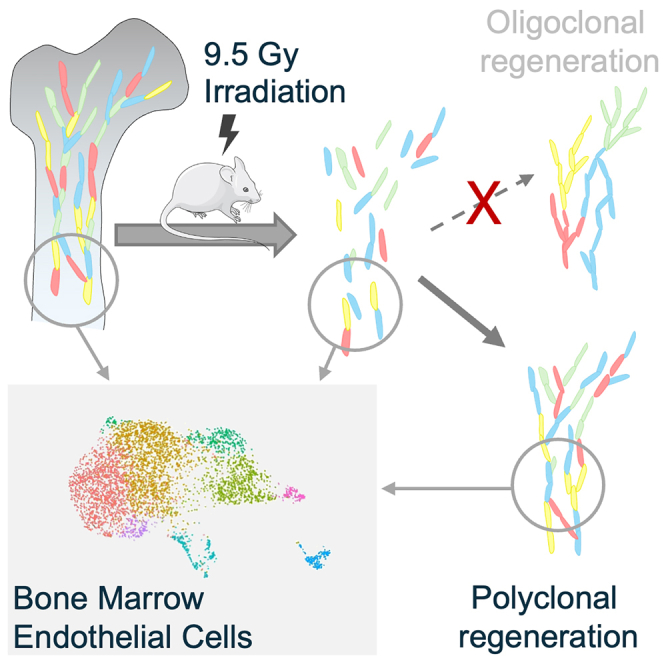

## Teaser

Irradiation, used for conditioning recipients before bone marrow (BM) transplantation, damages BM endothelial cells (BM-ECs). Skulimowska et al. elucidated the mechanisms behind BM-EC regeneration following irradiative conditioning. Their study revealed that a diverse population of BM-ECs can reenter the cell cycle and that the regeneration process is highly polyclonal.

## Introduction

Endothelial cells are a key component of the bone marrow (BM) microenvironment,^1^^,^^2^^,^^3^ particularly in providing a specialized niche for hematopoietic stem cells (HSCs).^4^^,^^5^^,^^6^^,^^7^ BM endothelial cells (BM-ECs) express adhesion molecules and produce factors necessary for the homing, differentiation, and self-renewal of HSCs.^8^^,^^9^ Consequently, the proper function of HSCs depends on BM-ECs, and disruption in BM-EC support leads to systemic hematopoietic collapse.^8^^,^^10^^,^^11^

While the role of BM-ECs in steady-state hematopoiesis is recognized, their function in post-hematopoietic cell transplantation (HCT) is less understood. Conditioning prior to HCT, involving chemotherapy and/or radiotherapy,^12^ negatively affects BM-ECs,^13^^,^^14^^,^^15^^,^^16^^,^^17^ making their regeneration essential for successful hematopoiesis reconstitution.

Some recent studies indicate the presence of local endothelial stem/progenitor cells driving the regeneration in the BM^17^ and other organs,^18^^,^^19^ while others suggest that BM-EC regeneration may result from a stochastic process characterized by significant EC plasticity.^20^^,^^21^^,^^22^^,^^23^^,^^24^^,^^25^^,^^26^^,^^27^

Understanding BM-EC regeneration may improve HCT outcomes, with therapeutic strategies potentially targeting progenitor populations or the signals regulating BM-EC plasticity.^28^^,^^29^

To investigate BM-EC regeneration post-irradiation, we analyzed mouse BM-EC heterogeneity using single-cell RNA sequencing (scRNA-seq) and identified candidate progenitor fractions. We further explored regeneration mechanisms and proposed a flow cytometry method to isolate key BM-EC fractions for functional assays. Our findings indicate significant polyclonal regeneration of the vascular network, with a significant subset of BM-ECs reentering the cell cycle.

## Results

### Heterogeneity of mouse BM-ECs in homeostasis

**Figure 1:**
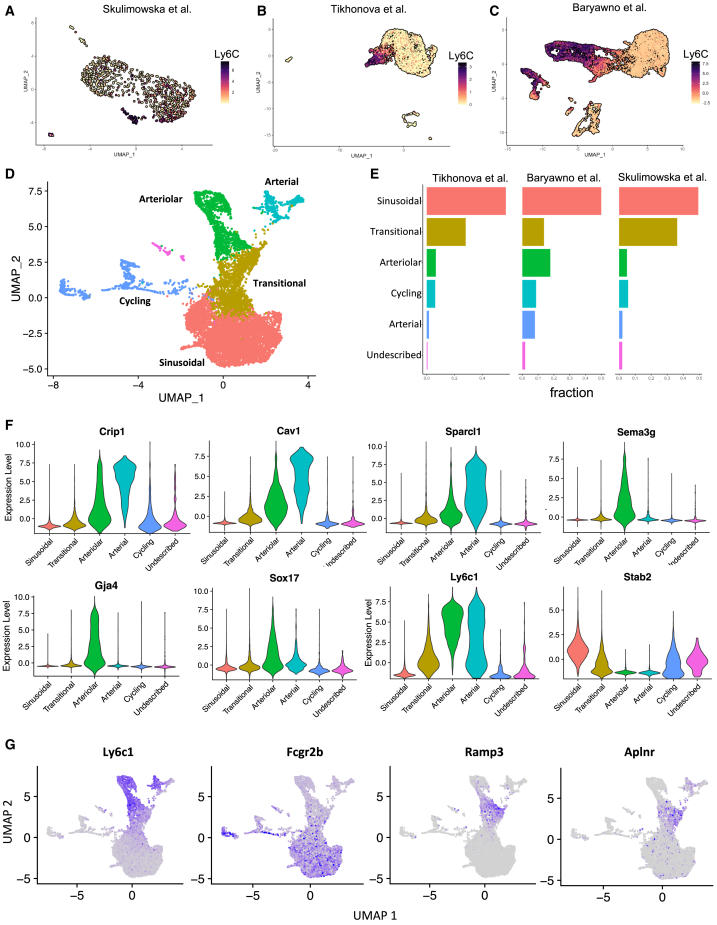
Combined meta-analysis of scRNA-seq data reveals the heterogeneity of the mouse BM-ECs (A–C) UMAP representation of scRNA-seq from mouse BM-EC data generated in this study (A) and in previous reports by Tikhonova et al. (B) and Baryawno et al. (C). Expression of Ly6c1 distinguishes types of ECs in three different datasets. For (A) and (B), log reads are shown, and for (C), data integrated with sctransform package (SCT) are shown. Data from Baryawno et al.^31^ constitute integrated data of ECs from 5 experiments. (D) Annotation of clusters identified within integrated data. (E) Cluster distribution in each dataset. (F and G) SCT-integrated expression of selected cluster markers in the integrated dataset.

To characterize the heterogeneity of mouse BM-ECs and identify potential endothelial progenitor subpopulations, we conducted plate-based scRNA-seq of sorted BM-ECs (CD45^−^Ter119^−^CD144^+^). Considering the impact of isolation methods on gene expression, we integrated our scRNA-seq data with previously published datasets, combining three independent studies.^30^^,^^31^ This meta-analysis minimized biases from different protocols and increased the number of analyzed cells, enhancing the resolution of BM-EC heterogeneity (Figures 1A–1C).

The analysis identified three main BM-EC types: sinusoidal, arteriolar, and arterial (Figure 1D). Data integration (see STAR Methods) ensured consistent cluster representation across all datasets, with no clustering by source (Figures 1D, 1E, and S1A). Slightly more cells from Baryawno et al. were found among arteriolar and arterial ECs (Figure 1E), aligning with prior annotations^31^ (Figure 1C).

Sinusoidal ECs were identified by high Stab2 and Fcgr2b expression^30^^,^^31^^,^^32^^,^^33^ (Figures 1F and 1G).

The distinction between arterial ECs and arteriolar ECs was based on the expression of genes such as Crip1, Cav1, Sparcl1, Lmna, and Vim, which are typical for large arteries and arterial ECs in other organs^34^ (Figures 1F and S1B). Genes expressed in the arteriolar cluster, such as Sema3g, Gja4, and Sox17 (Figures 1F and S1B), were shown to be present in both arterial and arteriolar capillaries in other organs^34^ and were consistently identified in previous analyses that distinguish arteriolar and arterial BM-ECs.^31^

The integration also revealed a “transitional” cluster, located between sinusoidal and arteriolar clusters, expressing markers from both (Fcgr2b, Ly6C) but lacking unique identifiers (Figures 1D–G, S1B, and S1C). However, Aplnr^17^^,^^35^^,^^36^^,^^37^^,^^38^^,^^39^ and Ramp3^34^ were overexpressed in this cluster, suggesting a potential role in angiogenesis (Figure 1G).

Additionally, cells enriched in genes linked to the cell cycle clustered separately (Figures 1D and S1D). Finally, we identified a small cluster of cells expressing endothelial markers, but their gene expression profiles did not allow for unequivocal annotation.

### Identification of BM-EC fraction during aging and after irradiation

**Figure 2:**
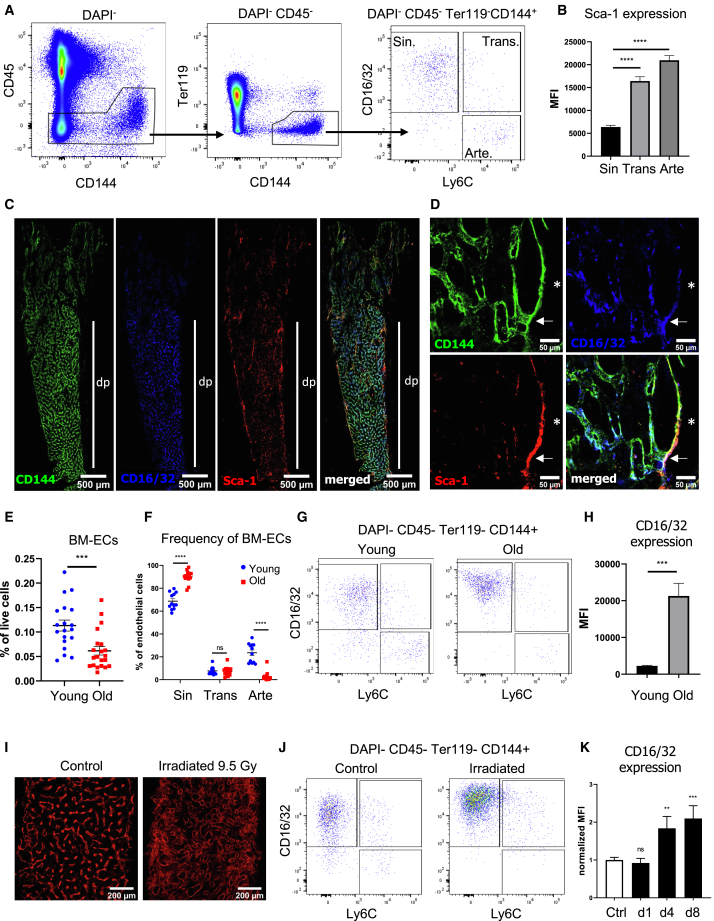
Prospective identification of BM-EC subtypes (A) Gating strategy of ECs in BM. ECs were stained by intravital injection of anti-CD144 antibody. CD16/32 and Ly6C antibodies distinguished three subpopulations, annotated as CD16/32^−^Ly6C^+^ arteriolar/arterial BM-ECs (Arte), CD16/32^+^Ly6C^+^ transitional BM-ECs (Trans), and CD16/32^+^Ly6C^−^ sinusoidal BM-ECs (Sin). (B) Flow cytometry analysis of Sca-1 (Ly6A) expression in BM-ECs. BM-ECs with high expression of Ly6C have also high expression of Sca-1 (Ly6A). MFI, mean fluorescence intensity. *n* = 6. Data are shown as mean ± SEM. ^∗∗∗∗^*p* < 0.0001, two-tailed unpaired t test. (C) BM-ECs in mouse femur. CD16/32^+^ vessels are located mainly in the diaphysis region and reveal sinusoidal morphology. dp, diaphysis. High Sca-1 expression characterizes the arteries and arterioles. (D) Sca-1/Ly6A^high^ CD16/32^−^ arterioles (^∗^) turn into transitional double-positive Sca-1/Ly6A^+^ CD16/32^+^ vessels (arrow), which gradually change to Sca-1/Ly6A^−/low^ CD16/32^+^ sinusoids. (E) Frequency of BM-ECs decreases with age. Three independent experiments, *n* = 19–21/group. Data are shown as mean ± SEM. ^∗∗∗^*p* < 0.001, two-tailed unpaired t test. (F) Old animals have a lower frequency of CD16/32^−^Ly6C^+^ arteriolar/arterial BM-ECs but a higher frequency of CD16/32^+^Ly6C^−^ sinusoidal BM-ECs among all BM-ECs. The fraction of transitional ECs does not change with age. Two independent experiments, *n* = 12–14/group. Data are shown as mean ± SEM. ^∗∗∗∗^*p* < 0.0001, two-tailed unpaired t test. (G) Representative flow cytometry plots show higher CD16/32 expression and frequency among BM-ECs in older animals. (H) Flow cytometry analysis of CD16/32 expression in sinusoids in young and old animals. MFI, mean fluorescence intensity. *n* = 6–7/group. Data are shown as mean ± SEM. ^∗∗∗^*p* < 0.001, two-tailed unpaired t test. (I) Irradiation affects the structure of blood vessels within the BM. Stained with intravital injection of anti-CD144 antibody. (J) Representative flow cytometry plots show higher CD16/32 expression in BM-ECs in irradiated animals (4 days after total body irradiation). (K) Flow cytometry analysis of CD16/32 expression in BM-ECs after total body irradiation. *n* = 3–8/group. Data are shown as mean ± SEM. ^∗∗∗^*p* < 0.001, two-tailed unpaired t test.

We prospectively identified and isolated the main fractions indicated by the scRNA-seq analysis. Using Ly6C and Fcgr2b (by CD16/CD32 antibody recognizing both Fcgr3 and Fcrg2b), we classified BM-ECs into arteriolar/arterial (Ly6C^+^CD16/32^−^), sinusoidal (Ly6C^−^CD16/32^+^), and transitional (Ly6C^+^CD16/32^+^) populations by flow cytometry (Figure 2A). However, due to Ly6C expression in abundant myeloid cells, we employed Ly6A (Sca-1) for microscopic analysis of BM-ECs’ populations, as it strongly correlates with Ly6C at both RNA (Figure S1E) and protein levels (Figures 2B and S1F). Microscopy of mouse tibia showed that transitional BM-ECs localize between arterioles and sinusoids, linking distinct EC types within the vascular network (Figures 2C, 2D, and S2). Transitional BM-ECs were present in both young (Figure S2A) and old mice (Figure S2B).

Next, we analyzed changes in BM-EC composition during aging and post-conditioning with 9.5 Gy irradiation and BM transplantation. BM-EC frequency decreases with age (Figure 2E), with old mice showing fewer arterial/arteriolar ECs (Ly6C^+^CD16/32^−^) but more sinusoids (Ly6C^−^CD16/32^+^) (Figures 2F and 2G). The frequency of transitional BM-ECs (Ly6C^+^CD16/32^+^) remains unchanged during aging (Figure 2F), but CD16/32 expression on sinusoidal ECs significantly increases in old mice (Figures 2G and 2H).

Finally, we assessed the impact of irradiation on the BM-EC phenotype. Seven days after 9.5 Gy irradiation, the BM vascular network appeared enlarged and diffused (Figure 2I). This was associated with a marked increase in CD16/32 expression on sinusoids at days 4 and 8 post-irradiation (Figures 2J and 2K). Thus, the expression of CD16/32 on sinusoids increases during aging and stress conditions.

### scRNA-seq reveals dynamic remodeling of BM-ECs after irradiative conditioning

To uncover the mechanisms of BM-EC regeneration, we conducted further scRNA-seq at 7, 21, and 60 days post-irradiation with 9.5 Gy (Figure S1G). A non-irradiated group processed alongside served as a control, ensuring high sensitivity in detecting molecular changes (Figure S1G).

**Figure 3:**
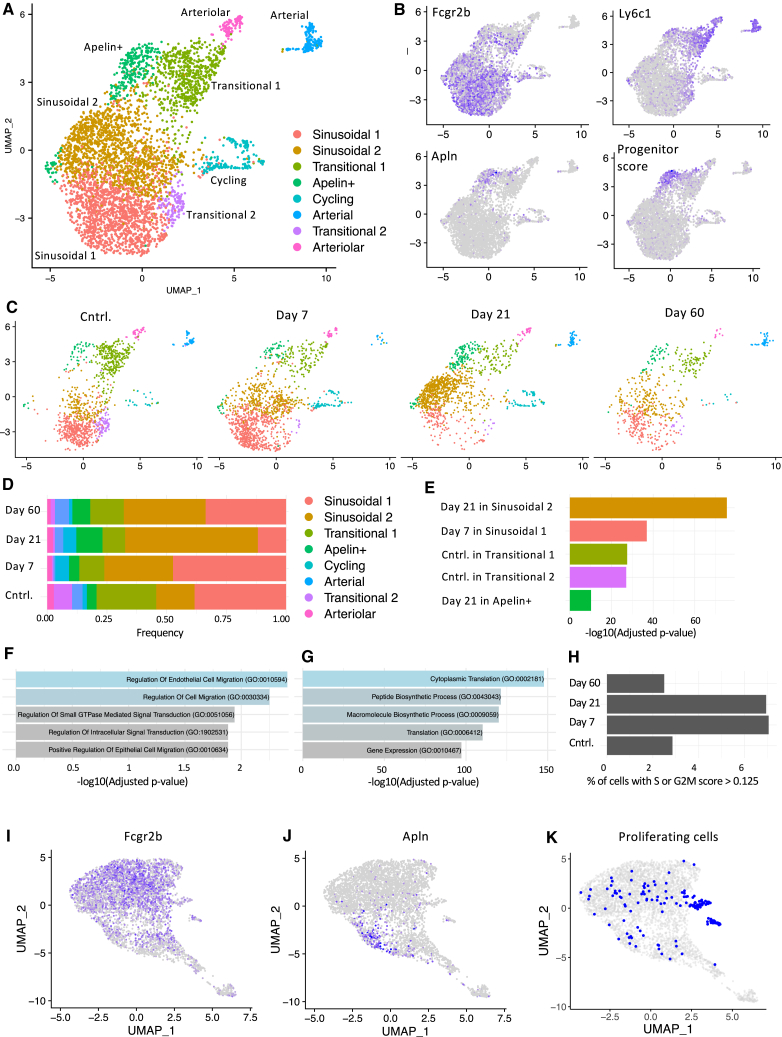
Single-cell RNA sequencing analysis reveals cellular and molecular alterations after irradiation (A) UMAP representation and identified clusters among combined non-irradiated and irradiated experimental groups. (B) Expression of Fcrg2b, Ly6c1, and Apln. The “progenitor score” reveals the combined signature score of selected genes typical for Apln^+^ EC progenitors in the lymph nodes.^19^ (C) UMAP representation of control cells and at days 7, 21, and 60 post-irradiation. (D) Cell distribution between clusters in control and irradiated groups. (E) Significant enrichment of cells from individual experimental group in identified clusters. Hypergeometric test with Bonferroni correction for multiple comparisons. (F) GSEA on marker genes of the sinusoidal cluster using the GO Biological Process (GOBP) database. (G) GSEA on differentially expressed genes between sinusoidal 2 and sinusoidal 1 clusters using GOBP database. (H) Frequency of cells among given experimental groups with high cell-cycling scores. (I–K) Novel UMAP projection after regressing the genes linked with S and G2M cell cycle phases, showing the expression of Fcgr2b and Apln, and highlighting the cells classified as cycling before regression.

The identified clusters (Figure 3A) matched those under homeostatic conditions (Figure 1D), including distinct arterial and arteriolar clusters. Genes specific for arterial (Tcf15, Meox2) and arteriolar (Gja4, Fbn1) cells aligned with previous analyses^31^ (Figure S3A). However, we also found three additional subclusters within transitional cells and two within sinusoidal cells.

Among transitional cells, we identified an Apelin-positive (Apln^+^) cluster, also characterized by Kit expression (Figures 3B and S3A). The Apln^+^ ECs were proposed as a population with progenitor potential that drive EC regeneration in different tissues.^17^^,^^19^ Based on scRNA-seq from Brulois et al.,^19^ we created a gene signature of Apln^+^ lymph node capillary progenitors that includes Apln, Kit, Nes, Sox4, Cd276, Cxcr4, Esm1, and Lxn genes (referred to as the “progenitor score”). The progenitor score signature was highly specific to the Apln^+^ cluster identified in our study (Figure 3B).

Another transitional cluster, termed transitional 2, expressed both sinusoidal and arteriolar markers (Ly6C^+^Fcrg2b^+^ phenotype) but not Ramp3 (Figure S3A). These cells clustered more closely with sinusoidal cells (Figure 3A). Transitional 1, resembling the original transitional cluster, expressed Ramp3 and high Cd34 levels (Figures 3A and S3A).

Finally, we also distinguished two clusters among sinusoidal cells (referred to as sinusoidal 1 and sinusoidal 2; Figure 3A). Although we did not find any unique markers for these subpopulations, the upregulation of several genes, including Rbfox1, Peak1, Adamts5, and Insr, distinguished the sinusoidal 2 cluster (Figure S3A). Analysis of BM-ECs distribution during regeneration (Figures 3C and 3D) revealed significant enrichment in sinusoidal 2 at day 21 and sinusoidal 1 at day 7 post-irradiation (Figure 3E). This indicates a transition in sinusoidal cells to a sinusoidal 2 profile between days 7 and 21.

Gene set enrichment analysis (GSEA) on sinusoidal 2 markers (200 genes with −log(*p*-adj) > 25) revealed the most upregulated terms “regulation of endothelial cell migration” and “regulation of cell migration” in the GO Biological Process database^40^^,^^41^ (Figure 3F) and the terms “focal adhesion” and “adherens junction” in the KEGG 2021 database^42^^,^^43^^,^^44^ (Figure S3B).

Moreover, GSEA based on differentially expressed (DE) genes between the sinusoidal 2 and sinusoidal 1 clusters (144 genes, with −log(*p*-adj) > 50) revealed high enrichment in terms indicating enhanced translation, biosynthetic processes, and gene expression in the GO Biological Process (Figure 3G) and Reactome^45^ (Figure S3C) databases. Altogether, at day 21 post-irradiation, sinusoidal BM-ECs displayed a transcriptional profile indicating active migration and high translation.

In contrast, both transitional 1 and 2 clusters were enriched in non-irradiated controls (Figure 3E), suggesting that the frequency of transitional BM-ECs is not fully restored post-irradiation (Figure 3D). Notably, the Apln^+^ cluster expanded significantly at day 21 (Figures 3D and 3E).

We then identified DE genes at days 7, 21, and 60 post-irradiation vs. controls and found the highest number of DE genes (with −log(*p*-adj) > 25) at days 21 (82 genes) and 7 (42 genes), with fewer at day 60 (6 genes) (Figure S3D). GSEA highlighted enrichment of the “p53 pathway,” “interferon gamma response,” and “apoptosis” at days 7 and 21 (Figures S3E and S3F) and interleukin (IL)-6/JAK/STAT3 signaling at day 60 (Figure S3G).

Next, using annotated cell cycle genes^46^ and cell cycle scoring in the Seurat package,^47^ we set thresholds on the obtained S and G2/M scores (Figure S3H) and estimated the number of cells in S or G2/M state. The highest frequencies of cycling cells were at days 7 and 21 post-irradiation (7.0% and 6.9%, respectively), with fewer cycling cells in controls and at day 60 (2.9% and 2.5%, respectively) (Figure 3H), which was in line with the percentage of cells in the cycling cluster (Figure S3J).

The distinct cell cycle gene expression program clustered cycling cells separately, obscuring their BM-EC origin (Figure 3A). To overcome this, we regressed out cell cycle-related genes using the scTransform approach,^48^ followed by new data scaling, dimension reduction, and uniform manifold approximation and projection (UMAP) representation (Figures 3I–3K). The new UMAP representation resembles the original one, including the clustering of Apln^+^ cells (Figures 3I and 3K). Although the regression did not completely remove the separate clustering of cycling cells, some were reclassified into sinusoidal and transitional clusters (Figure 3K), indicating that these BM-EC types can enter the cell cycle (Figure 3K).

### BM-ECs exhibit high clonogenic potential *in vitro*

**Figure 4:**
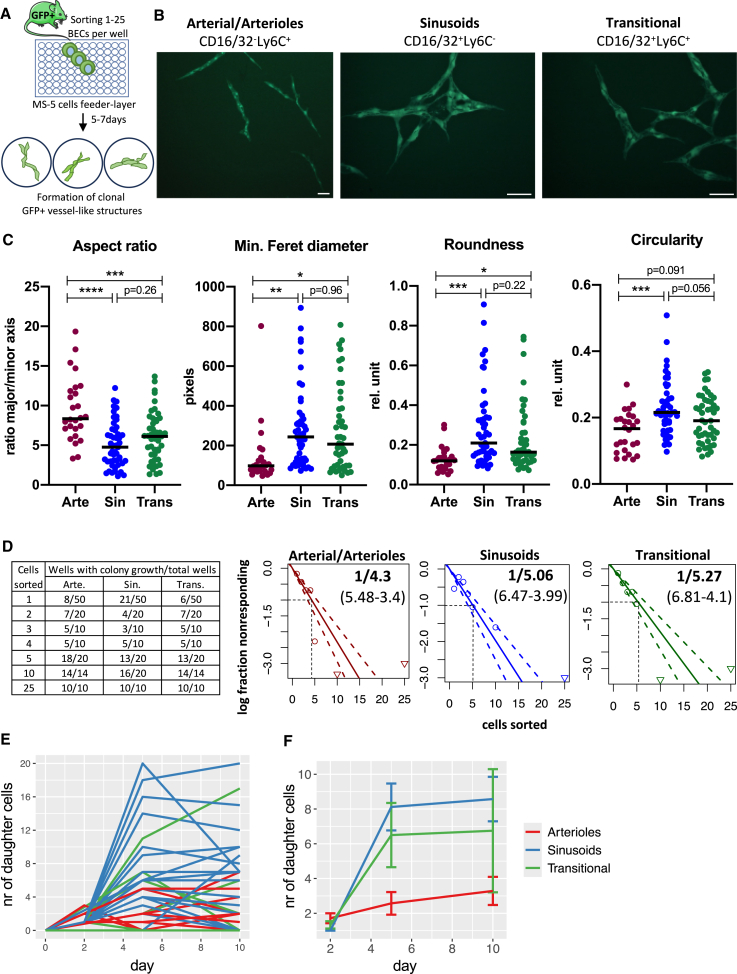
Colony *in vitro* assay shows high clonogenicity of mouse BM-ECs (A) Scheme of the single-cell colony assay designed to study clonogenic potential of BM-ECs. (B) Morphology of the growing vessel-like structures 7 days after sorting different subpopulations of BM-ECs. Bars indicate 200 μm. (C) Quantitative analysis revealed morphological differences of vessel-like structures derived from different subpopulations of BM-ECs. *n* = 26–49/group, two-tailed unpaired t test, ^∗^*p* < 0.05, ^∗∗^*p* < 0.01, ^∗∗∗^*p* < 0.001, and ^∗∗∗∗^*p* < 0.0001. (D) Limiting dilution assay demonstrated high *in vitro* clonogenic potential of BM-ECs regardless of the analyzed subpopulation. Triangles show data points beyond the scale of the graph. The dotted line and values in brackets reflect 95% confidence interval (CI). (E) Number of cells derived from single sorted cells from different subpopulations of BM-ECs. (F) Mean value with SEM of cell output from single sorted cells from different subpopulations of BM-ECs, total *n* = 14–45/group.

Irradiation causes significant damage to the vascular niche in BM.^13^^,^^14^^,^^15^^,^^16^^,^^17^ To determine if any BM-EC subpopulation has clonogenic potential for vascular regeneration, we developed an *in vitro* clonogenic assay. We sorted sinusoidal, arteriolar/arterial, or transitional BM-ECs from GFP-expressing mice over a feeder layer of MS-5 mesenchymal cells (Figure 4A). After 5–7 days, sorted cells formed GFP^+^ vessel-like structures (Figure 4B) with different morphologies. Arteriolar/arterial ECs developed into more elongated and spindle-like shapes, while sinusoidal cells had higher circularity and roundness (Figure 4C), resembling, at least partially, the morphological differences between arterioles and sinusoids in the BM niche. Transitional BM-ECs resembled sinusoidal BM-ECs rather than arteriolar/arterial BM-ECs (Figure 4C).

To quantify the clonogenicity of the three BM-EC fractions we performed a limiting dilution assay^49^ (LDA) (Figure 4D). All BM-EC fractions gave rise to colonies and had high clonogenic potential (1 out of 4.3 arteriolar/arterial, 1 out of 5.06 sinusoidal, and 1 out of 5.23 transitional sorted cells), with no significant differences between groups (Figure 4D). This indicates that a substantial fraction of any of the main BM-EC types can reenter the cell cycle.

We then counted the daughter cells from single-cell-sorted BM-ECs. Cells divided until day 5, but numbers plateaued from day 5 to day 10, followed by structural disintegration (Figure 4E). Sinusoidal and transitional cells produced more daughter cells (8.6 ± 1.4, 6.8 ± 3.5) than arteriolar/arterial BM-ECs (3.3 ± 0.8) (Figure 4F).

### Polyclonal regeneration of BM-ECs after irradiation and BM transplantation

**Figure 5:**
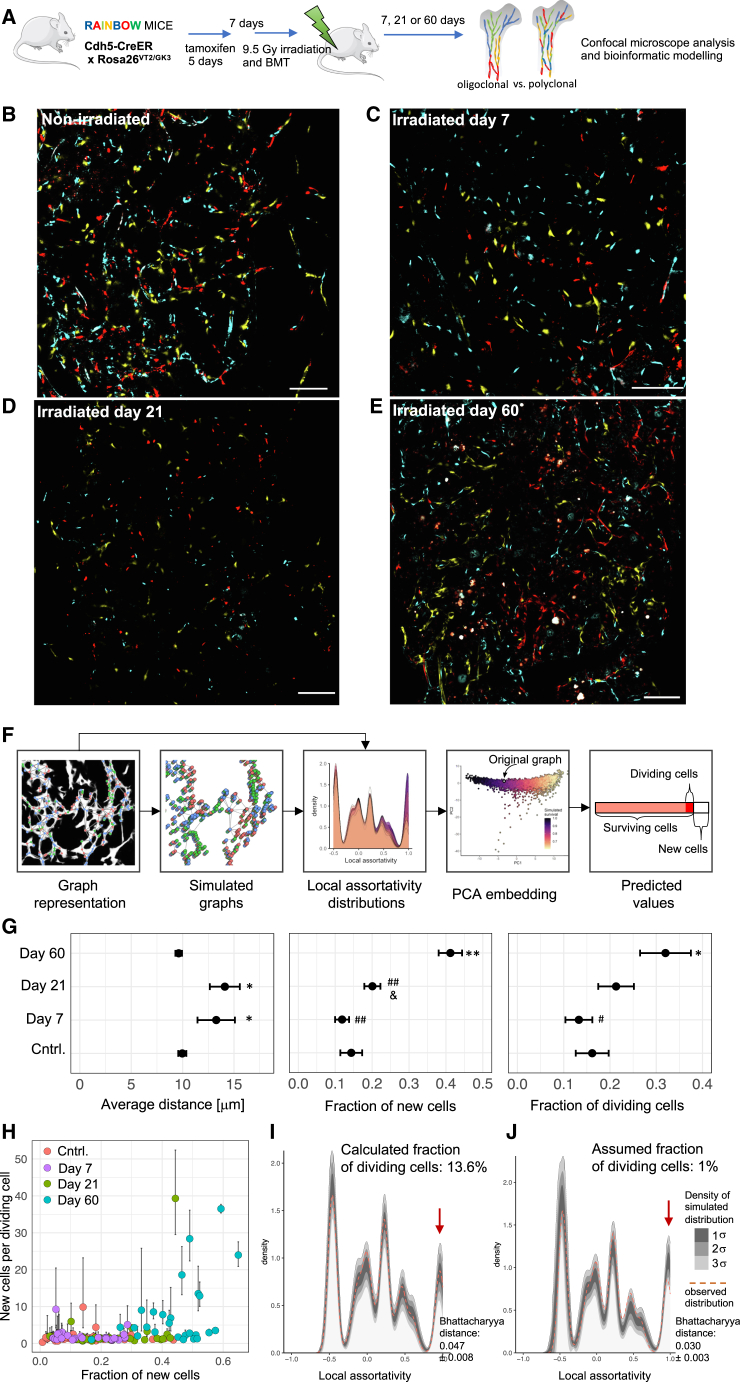
Polyclonal regeneration of BM-ECs after irradiative conditioning and BM transplantation in mice (A) Experimental scheme used to evaluate the clonality of BM-EC regeneration *in vivo*. (B–E) Expression pattern of Rainbow fluorescent proteins in non-irradiated and irradiated BM. White: VE-cadherin, blue: mCerulean, yellow: mOrange, and red: mCherry. Bar indicates 100 μm. (F) Scheme of the bioinformatic pipeline used to quantify the regeneration. (G) Estimated parameters describing the regeneration of the mouse BM-ECs. In the model, the total number of 48,675 cells was included within 138 graphs derived from images from 20 mice, *n* = 3–6 mice per group. The error bars represent the error propagated during each modeling step.^∗^*p* < 0.05 vs. control (cntrl.); ^∗∗^*p* < 0.01 vs. cntrl.; *p* < 0.05 vs. day 7; #*p* < 0.05 vs. day 60; and ##*p* < 0.01 vs. day 60. (H) Estimated number of daughter cells derived from the first dividing cell depending on the fraction of new cells generated after irradiation. (I and J) The observed distribution of local assortativity of representative graph (dotted orange line) overlayed over 500 simulations (shown as density). In (I), the simulations were based on best-fitted value of the fraction of dividing cells for each individual graph (13.6%), while in (J), we assumed a 1% fraction of dividing cells. The red arrow in (G) shows that the potential presence of rare 1% progenitors would be manifested within the high local assortative distribution values (here visible as the discrepancy between simulations and observed distribution).

We investigated whether the high clonogenic potential of BM-ECs observed *in vitro* contributes to the regeneration of BM-ECs after irradiation and BM transplantation. For this purpose, we used Rainbow mice (Cdh5-CreER; Rosa26^VT2/GK3^), which randomly express fluorescent proteins in ECs upon tamoxifen induction. One week after induction, we performed irradiation (9.5 Gy), transplanted BM, and analyzed the distribution of fluorescent labeling among BM-ECs 7, 21, and 60 days post-irradiation (Figure 5A).

In non-irradiated mice, recombination was induced specifically in the ECs, showing random distribution of the three fluorophores in diaphysis and metaphysis regions (Figures 5B, S4A, and S4B). We did not observe single-color regions within BM-ECs at day 7 or 21 after irradiation (Figures 5C, 5D, and S4C). However, by day 60 post-irradiation, we detected regions of vessels that appeared to be non-randomly formed by single-colored BM-ECs (Figures 5E and S4C).

To quantify the color distribution and clonality, we developed a method based on graph theory, local assortativity, and machine learning (Figure 5F; STAR Methods). First, using U-Net convolutional deep neural networks, we represented the BM-EC network as a series of graphs, with the BM-ECs as the nodes (each graph was modeled independently; Figure 5F), and trained the machine learning algorithm through iterative simulations. We simulated different percentages of BM-EC death during irradiation and, among the surviving BM-ECs, different percentages undergoing cell division and clonal expansion to compensate for BM-EC loss. We used parameters of local assortativity distribution within the graphs to represent color distribution—areas with the same fluorochrome have high assortativity, while areas with random fluorochrome expression have low assortativity. The trained model, validated with simulated data, was then applied to experimental data (STAR Methods).

The graph representation highlighted the impact of irradiation on the BM-EC network and tracked its regeneration over time. The model indicated increased average distances between BM-EC centroids at days 7 and 21 post-irradiation (13.2 ± 1.8 and 14.1 ± 1.5 μm, respectively) compared to non-irradiated mice (10.0 ± 0.1 μm, *p* < 0.05; Figure 5G), showing incomplete regeneration. By day 60, distances were similar to controls (9.61 ± 0.32 μm), suggesting that the vascular network structure had normalized (Figure 5G).

Next, we estimated the fraction of new BM-ECs, defined as the percentage of BM-ECs at a given time point (day 7, 21, or 60 post-irradiation) that were generated from the time of tamoxifen induction. Compared to the non-irradiated group, the increase in new cells at 7 days post-irradiation was not significant (11.9% ± 1.9%, *p* = 0.35). However, by day 21, it rose to 20.0% ± 2.9% (*p* < 0.05 vs. day 7), reaching 41.2% at day 60 (*p* < 0.01 vs. control and day 7; Figure 5G). This indicates that irradiation significantly enforces BM-EC turnover, though regeneration began after a delay, was still active at day 21, and continued for up to 60 days.

Our model allowed us to estimate the fraction of surviving BM-ECs that underwent cell division(s), which indicates the initial frequency of BM-EC progenitors. 32.0% ± 5.5% of BM-ECs surviving irradiation entered the cell cycle and contributed to new BM-ECs by day 60 (*p* < 0.05; Figure 5G), indicating a highly polyclonal regeneration.

We also estimated the number of daughter cells produced by each proliferating cell. On average, dividing cells produced 1.9 ± 0.3 daughter cells at day 7, 2.6 ± 1.0 at day 21, and 8.0 ± 1.8 by day 60 (Figure 5H). At day 60, we observed a subset of high-output cells generating 10–40 daughter cells, particularly in vessel fragments with the highest fraction of new cells.

Finally, we tested our model’s sensitivity to detect clonal expansion in potentially rare BM-EC progenitor fractions. We analyzed exemplary graphs and overlaid the observed distribution of local assortativity with 500 simulations. The simulations used the best-fitted values of dividing cells (Figure 5I, best-fitted value, 13.6% for the graph) or assumed the presence of a rare 1% fraction of dividing progenitors (Figure 5J). If rare clonal expansion existed, then we would see high local assortativity peaks (red arrows, Figure 5J), but such patterns were not observed and are not supported by the obtained data.

Overall, our model suggests that BM-EC regeneration after irradiation is polyclonal, driven by a broad fraction of BM-ECs, though cell output varies among clones in later regeneration stages.

## Discussion

Our study revealed the cellular mechanism of BM-EC regeneration. The data indicate that a broad fraction of BM-ECs can reenter the cell cycle and provide polyclonal regeneration of BM vasculature after irradiation. A prospective LDA assay showed that both arterioles and sinusoids in the BM can proliferate and undergo at least a few cell divisions (Figure 4). Using scRNA-seq, we identified a transitional subpopulation of BM-ECs expressing both sinusoidal and arteriolar markers, including the Apln receptor (Aplr), associated with angiogenic stalk ECs in other organs (Figures 1D and 1G).^35^ Among this transitional phenotype, some cells express Apln, which is proposed as a marker of an endothelial progenitor population in lymph nodes and BM^17^^,^^19^ (Figures 3A and 3B).

However, we did not observe enhanced clonogenic potential in the transitional population compared to sinusoidal or arterial cells in the LDA *in vitro* assay (Figure 4D). Analysis of BM-EC clonality *in vivo*, after irradiation and BM transplantation, showed that by 60 days post-irradiation, 32.0% of surviving BM-ECs reentered the cell cycle, producing an average of 8 daughter cells (Figure 5G). This number closely matches the average number of cells obtained *in vitro* from single sorted sinusoidal (8.6 ± 1.4) or transitional (6.8 ± 3.5) cells (Figure 4F). Therefore, the data do not support the contribution of a rare, clonally expanding endothelial fraction in BM-EC regeneration after irradiation and BM transplantation. Instead, regeneration appears polyclonal, driven by a broad fraction of cells. However, we did observe fragments of vessels (up to 40 cells) originating from a single cell, indicating some BM-ECs have higher clonal expansion potential.

Our study provides a detailed characterization of the impact of irradiation on BM-ECs and the course of their regeneration and highlights underlying cellular and molecular mechanisms at the single-cell level (Figures 3 and S3). At day 7 post-irradiation, there is no regeneration of cell loss (Figure 5G), but cells begin to proliferate (Figure 3H). Day 21 is the most active phase of regeneration, with a significant shift in the transcriptional profile of sinusoidal BM-ECs, suggesting active migration and enhanced translation (Figures 3F and 3G). However, cell loss is not yet compensated (Figure 5G). The vascular network structure is restored by day 60 (Figure 5G), though some transitional clusters have not reached their initial frequency (Figures 3D and 3E). The gene expression profile of BM-ECs at 60 days post-irradiation resembles that of non-irradiated cells. Importantly, over 40% of BM-ECs at day 60 are newly generated, highlighting the impact of irradiation on BM-ECs.

Studies on ECs in other organs also highlight EC plasticity in response to injury. Endothelial regeneration of the aorta is driven by differentiated ECs reentering the cell cycle.^22^^,^^23^ Myocardial infarction induces a proliferative state in myocardial ECs,^26^ and stochastic phenotype switching is observed in many vascular beds, supporting general EC plasticity.^20^

However, EC regeneration after injury may differ from new blood vessel sprouting.^22^ Sprouting angiogenesis employs distinct genetic programs from regeneration of ECs after injury,^22^^,^^50^ with a well-documented role for Apln signaling.^35^ Neo-vascularization in lymph nodes during inflammation is driven by Apln^+^ endothelial progenitors.^19^ Moreover, previous study proposed that proliferating ECs in the BM after irradiation represent an Apln-expressing population.^17^ We observed a corresponding Apln^+^ BM-EC cluster among transitional cells in one of our datasets, which closely shares the marker gene signature of lymph node Apln^+^ progenitors (Figure 3B). However, our data suggest that BM-EC regeneration post-irradiation does not solely depend on rare Apln^+^ progenitors (4% of BM-ECs in non-irradiated mice; Figure 3D). Our model indicates a polyclonal regeneration, with over 30% of initial BM-ECs entering the cell cycle. We also showed that sinusoids, the most common BM-EC type, have a high potential to reenter the cell cycle (Figure 4D). Additionally, our scRNA-seq data indirectly show that cells with high cell-cycling scores are found among sinusoids and transitional cells. Previous histological studies confirmed that some sinusoidal cells in the BM after irradiation display cell-cycling markers.^16^

Nonetheless, we observed a few larger single-cell-derived clones (up to 40 cells) forming small vessel fragments. These could be derived from Apln^+^ progenitors, while broader BM-EC loss is polyclonally regenerated by local sinusoidal cells. However, since we induced Rainbow labeling before irradiation in all BM-ECs, our model cannot address this question.

Our study introduces prospective and quantitative approaches to study BM-EC clonogenic potential. We developed an assay showing that prospectively isolated sinusoidal, arteriolar, and transitional BM-ECs can reenter the cell cycle at the single-cell level. Additionally, we propose a method to analyze BM-EC regeneration using Rainbow mice. This approach avoids the drawback of leakiness found in reporter gene-driven systems. Our method employs graph theory and a machine learning model trained with various regeneration patterns, allowing us to uncover clonality mechanisms quantitatively (available as an R package; see STAR Methods). We also describe the heterogeneity of mouse BM-ECs using our and two other scRNA-seq datasets, reducing the impact of isolation methods and sequencing technology differences, representing a comprehensive single-cell atlas of mouse BM-ECs (publicly available at https://morys.shinyapps.io/complete_RNAseq/). Our scRNA-seq dataset on BM-EC regeneration post-irradiation provides a unique resource for identifying new molecular mechanisms and therapeutic targets (publicly available at https://bmecs-atlas.szadelab.bio.edu.pl:3838/).

In conclusion, our study reveals a polyclonal contribution of BM-ECs to BM niche vasculature regeneration post-irradiation. Given the critical role of BM-EC regeneration in BM niche recovery for successful HSC engraftment, further research should focus on the molecular mechanisms that regulate BM-EC plasticity and cell cycle reentry under stress conditions.

### Limitations of the study

The limitation of this study is that our model does not account for how the heterogeneity and function of regenerating ECs change over time. It is possible that the regeneration of BM-ECs comprises different phases, each varying in clonality and contributing differently to the final number of new BM-ECs. In our approach, we label BM-ECs and their progeny before irradiation and end the analysis at different time points, which provides us with average information about the clonality parameters of the process over a given period after irradiation. However, we cannot exclude the possibility that during the analyzed time frame, there were distinct phases of regeneration with differing clonality parameters. Future research could address this limitation using *in vivo* barcoding-based lineage tracking methods coupled with spatial single-cell transcriptomics.

## Resource availability

### Lead contact

Further information and requests for resources and reagents should be directed to and will be fulfilled by the lead contact, Krzysztof Szade (krzysztof.szade@uj.edu.pl).

### Materials availability

• This study did not generate new unique reagents.

### Data and code availability

• Data and visualization of scRNA-seq analyses are publicly available as follows: at https://morys.shinyapps.io/complete_RNAseq/ for integrated datasets of BM-ECs in steady state and https://bmecs-atlas.szadelab.bio.edu.pl:3838/ for the scRNA-seq Atlas of Mouse Bone Marrow Endothelial Cell Regeneration Post-Irradiation (Day 7, 21, and 60). The raw sequence files are deposited in the European Nucleotide Archive (ENA:PRJEB76309).
• Code for cell network analysis can be found here: https://github.com/jmorys/RainbowGraph.
• Any additional information required to reanalyze the data reported in this work is available from the lead contact upon request.

## Acknowledgments

The study was founded by the Foundation of Polish Science within the “Homing” programme (POIR.04.04.00-00-5F16/18-00), ERC Starting Grant “StemMemo” (101041737) granted to K.S., and by the National Science Center within the “Maestro” programme granted to A.J. (2018/30/A/NZ3/00495). A.S. received the L’Oreal-UNESCO For Women in Science Award. M.G. was supported from Budgetary Funds for Science in 2020-2022 (Diamond Grant 0102/DIA/2020/49). The research and open access publication has been supported by grants from the Priority Research Area BioS and the Faculty of Biochemistry, Biophysics and Biotechnology (FBBB) under the Strategic Programme Excellence Initiative at Jagiellonian University (JU). We would like to acknowledge the Animal Facility Staff (FBBB) at the Genomics Core Facility Małopolopska Center of Biotechnology JU.

## Author contributions

I.S., J.M., J.S., M.G., R.S., G.G., K.K., S.M., A.S., and K.S. performed experiments and analyzed data. J.M. designed the model to analyze clonality based on the color distribution in Rainbow mice. J.M. and K.S. performed bioinformatic analysis. A.S., I.L.W., A.J., and K.S. planned and supervised the study. I.S., J.M., A.S., and K.S. wrote the manuscript.

## Declaration of interests

The authors declare no competing interests.

## Declaration of generative AI and AI-assisted technologies in the writing process

During the preparation of this work, the authors used ChatGPT in order to check and correct language and readability. The authors reviewed and edited the content as needed and take full responsibility for the content of the publication.

## STAR★Methods

### Key resources table

**Table:** 

REAGENT or RESOURCE	SOURCE	IDENTIFIER
**Antibodies**		

CD144 – Alexa Fluor 647 (clone BV13)	BioLegend	Cat# 138006; RRID: AB_10568319
CD45 – Brilliant Violet 786 (clone 30-F11)	BD Biosciences	Cat# 564225; RRID: AB_2716861
CD45 – APC-Cy7 (clone 30-F11)	BioLegend	Cat# 103116; RRID: AB_312981
Ter119 – APC-Cy7 (clone TER119)	BD Biosciences	Cat# 560509; RRID: AB_1645230
Ter119 – PE-Cy7 (clone TER119)	BD Biosciences	Cat# 557853; RRID: AB_396898
CD16/32 – PE (clone 2.4G2)	BD Biosciences	Cat# 561727; RRID: AB_10892816
CD16/32 – Brilliant Violet (clone 2.4G2)	BD Biosciences	Cat# 752948; RRID: AB_2917903
CD16/32 – BUV395 (clone 2.4G2)	BD Biosciences	Cat# 740217; RRID: AB_2739965
Ly6C – PerCP-Cy5.5 (clone HK.1.4)	BioLegend	Cat# 128012; RRID: AB_1659241
Ly6A/E (Sca-1) – PE-Cy7 (clone D7)	BD Biosciences	Cat# 558162; RRID: AB_647253
Ly6A/E (Sca-1) – unconjugated (polyclonal goat IgG)	R&D Systems	Cat# AF1226; RRID: AB_354679
Donkey anti-Goat IgG (H + L) Cross-Adsorbed Secondary Antibody – Alexa Fluor 594 (polyclonal)	Thermo Fisher Scientific	Cat# A-11058; RRID: AB_2534105
Hashtag 1 - TotalSeq-B0301 (clone M1/42; 30-F11)	BioLegend	Cat# 155831; RRID: AB_2750032AB_2814067
Hashtag 2 - TotalSeq-B0302 (clone M1/42; 30-F11)	BioLegend	Cat# 155833; RRID: AB_2750033AB_2814068
Hashtag 5 – TotalSeq-B0305 (clone M1/42; 30-F11)	BioLegend	Cat# 155839; RRID: AB_2750036AB_2814071
Hashtag 6 - TotalSeq-B0306 (clone M1/42; 30-F11)	BioLegend	Cat# 155841; RRID:AB_2750037AB_2814072

**Chemicals, peptides, and recombinant proteins**		

Tamoxifen	Sigma-Aldrich	Cat# T5648-1G
DAPI	Sigma-Aldrich	Cat# D9542-10MG
Collagenase, Type IV, powder	Thermo Fisher Scientific	Cat# 17104019
Collagenase, Type I, powder	Thermo Fisher Scientific	Cat# 17100017
Dispase II, powder	Thermo Fisher Scientific	Cat# 17105041
DNase I from bovine pancreas	Merck	Cat# 11284932001
Paraformaldehyde solution 4% in PBS	Santa Cruz Biotechnology	Cat# sc-281692
Iso-pentane (2-Methylbutane)	VWR Chemicals	Cat # 103616V
EDTA disodium salt dihydrate, powder	POCH	Cat# 879810112

**Critical commercial assays**		

Live/dead Fixable Near-IR Dead Cell Stain Kit, for 633 or 635 nm excitation	Thermo Fisher Scientific	Cat# L34976
Live/dead™ Fixable Yellow Dead Cell Stain Kit, for 405 nm excitation	Thermo Fisher Scientific	Cat# L34967

**Deposited data**		

Interactive web atlas of scRNA-seq data	https://bmecs-atlas.szadelab.bio.edu.pl:3838/	N/A
Raw scRNA-seq data	ENA:PRJEB76309	N/A
Code for cell network analysis	https://github.com/jmorys/RainbowGraph	N/A

**Experimental models: Cell lines**		

MS-5 (murine stromal cells)	DSMZ	Cat# ACC 441

**Experimental models: Organisms/strains**		

Mouse: C57BL6/J	In house breeding	N/A
Mouse: C57BL6-Rainbow(R26 VT2/GK3)/Ilw x C57BL6(Cdh5-CreER)/Ilw	In house breeding	Derived from RRID: MGI:5441200
Mouse: C57BL/6-Tg(UBC-GFP)30Sch/J	In house breeding	RRID:IMSR_JAX:004353

**Software and algorithms**		

FACSDiva	BD Biosciences	https://www.bdbiosciences.com/en-ca/products/software/instrument-software/bd-facsdiva-software
FlowJo v 10.8.1	BD Biosciences	https://www.flowjo.com/
Prism v 8	GraphPad	https://www.graphpad.com/scientific-software/prism/
Microsoft Excel	Microsoft	https://www.microsoft.com/en-gb/
ImageJ (FIJI)	Schneider et al. 2012^51^	https://imagej.nih.gov/ij/
Python	Van Rossum et al. 2009^52^	https://www.python.org/
NumPy	Harris et al. 2020^53^	https://numpy.org/
scikit-image	Van der Walt et al.^54^	https://scikit-image.org/
Pytorch	Paszke et al. 2019^55^	https://pytorch.org/
Scipy	Virtanen et al. 2020^56^	https://scipy.org/
scikit-fmm	N/A	https://github.com/scikit-fmm/scikit-fmm
Matplotlib	Hunter 2007^57^	https://matplotlib.org/stable/
R (v 4.4.0)	https://www.R-project.org/	https://www.R-project.org
Tidyverse	Wickham et al. 2019^58^	https://www.tidyverse.org/
Reticulate	Ushey et al. 2024^59^	https://rstudio.github.io/reticulate/
Igraph	Csardi and Nepusz^60^	https://igraph.org/
Parallel	N/A	https://cran.r-project.org/web/packages/foreach/index.html

### Experimental model and study participant details

#### Animals

All animal procedures and experiments were performed in accordance with national and European legislations, after approvals (40/2020, 150/2020, 325/2020, 229/2022,185/2023) by the Second Local Ethical Committee on Animal Testing in Krakow.

The 2- and 23-month-old female C57BL6/J mice were used for BM-ECs frequency analysis during aging. The 3- and 19-month-old male C56BL6/J mice were used for IHC staining. The 3-month-old male and female C57BL6/J mice were used for studying BM-ECs frequency after total body irradiation. The 2-month-old male C57BL6-Rainbow(R26 VT2/GK3)/Ilw x C57BL6(Cdh5-CreER)/Ilw mice were used for bone marrow vasculature regeneration analysis after BMT. In the limited dilution assay (LDA) the BM-ECs isolated from 2-month-old male C57BL/6-Tg(UBC-GFP)20SchJ mice were used. Both males and females were used in the study and we did not find any influence of sex in experiments where females and males were directly compared.

### Method details

#### Recombination induction

To induce Cre recombinase expression in endothelial cells mice were administered with tamoxifen (Sigma-Aldrich, dissolved in corn oil at concentration of 20 mg/mL) by intraperitoneal injections at 75 mg/kg body weight for 5 consecutive days.

#### Irradiation and BM transplantation

The mice underwent whole-body irradiation using either Cesium-137 gamma-rays or X-rays, performed 7 days after the last dose of tamoxifen. The mice were positioned in a mouse-pie cage and irradiated with a single dose of 9.5 Gy. After 24 h, mice were transplanted with 2 x 10^6^ mononuclear bone marrow cells isolated from 3-month-old C57BL6/J donor. Transplantation was carried out via retro-orbital injection. Mice were sacrificed at 7, 21, or 60 days post-irradiation.

#### Isolation of BM-ECs

To stain BM-ECs with the anti-CD144 antibody, mice were anesthetized, and 12.5 μg of the antibody was injected into the retro-orbital sinus. After 10 min, the mice were euthanized, and hindlimb bones were isolated. The bones were then cut into small fragments and suspended in an enzyme mix containing 1.5 mg/mL Collagenase type I, 1.5 mg/mL Collagenase type IV, 3.0 mg/mL Dispase II, and 25 μg/mL DNAse type I in PBS with calcium and magnesium. The digestion was carried out at 37°C for 15 min and repeated three times with a fresh portion of the enzyme mix each time. Following digestion, red blood cells were lysed using RBC Lysis Buffer (155 mM NH_4_Cl, 14 mM NaHCO_3_, 1 mM EDTA in ddH_2_O) for 5 min at room temperature. Subsequently, *ex vivo* surface staining was performed.

#### Flow cytometry and FACS sorting

Flow cytometry analysis was done on LSRFortessa cytometer (BD Biosciences). Cell sorting was done on MoFlo XDP cells sorter (Beckman Coulter) or Bigfoot Spectral Cell Sorter (Thermo Fisher Scientific). Fluorescently labeled antibodies used in this study were purchased from BD Biosciences and BioLegend. All antibodies are listed in key resources table. The cells were stained in FACS buffer (2% (v/v) FBS in PBS) for 30 min at 4°C in the dark. Prior to flow cytometry analysis or cell sorting the cells were filtered via 40μm cell strainer. The populations used in studies were defined as follows: BM-ECs – CD45^−^Ter119^−^CD144^+^, S-ECs – CD45^−^Ter119^−^CD144^+^CD16/32^+^Ly6C^−^, A-ECs - CD45^−^Ter119^−^CD144^+^CD16/32^−^Ly6C^+^, Transitional-ECs - CD45^−^ Ter119^−^ CD144^+^ CD16/32^+^ Ly6C^+^.

#### Limited dilution assay

MS-5 cells were seeded 24 h before the assay at a density of 10,000 cells per well in MEM alpha medium (Gibco) supplemented with 10% (v/v) FBS and 1% (v/v) penicillin/streptomycin (Gibco). The following day, the medium was changed to endothelial growth medium (EGM-2MV Microvascular Endothelial Cell Growth Medium-2 BulletKit, Lonza) supplemented with 10% (v/v) FBS and 1% penicillin/streptomycin. Next, BM-ECs were isolated from C57BL/6-Tg(UBC-GFP)20SchJ mice, which exhibit ubiquitous expression of GFP, and 1–25 cells were sorted per well onto the MS-5 layer. Over the next 10 days, wells were screened for the presence of GFP^+^ vessel-like structures, with media changes performed every 3–4 days. Photographs of each vessel-like structure were captured using a fluorescence microscope (Nikon Eclipse Ti), and the cell shapes were delineated using FIJI Software in blinded fashion. Quantitative analysis of morphology was conducted using FIJI Software.

#### Immunohistochemistry

Tibias and femurs were fixed in fixation buffer (4% PFA with 10% EDTA and 0.25% Triton X-100, pH 7.2–7.4) for 8–20h on ice in 4°C while rotating, followed by overnight incubation in 20% EDTA in 4°C (pH 7.2–7.4). Next, the bones were sequentially incubated in sucrose solutions in PBS: 10%—1h, 20%—1h, and 30%—overnight in 4°C. After removing excess sucrose, the bones were embedded in OCT Tissue Freezing Medium (Leica) and frozen in dry-ice-cooled isopentane (VWR Chemicals). 70-μm-thick longitudinal section of bones were cut on cryostat (Leica), blocked with blocking medium (10% donkey serum, 1% BSA, 0.3M glycine, 0.05% Triton X-100) and stained overnight with anti-Sca-1 primary antibody (polyclonal goat, R&D Systems) at 4°C, followed by 2h staining with secondary antibody (donkey anti-goat AF594, Thermo Fisher Scientific) at room temperature. CD144 and CD16/32 staining was performed *in vivo* as described before. Imaging of whole bone fragments was done on LSM780 confocal microscope (Zeiss) across three different focal planes (within ±10 μm), using 10× or 20× objective, resulting in approximately 100–200 images in five channels per bone. These images were stitched together, and maximum intensity projections were created to produce the final image of the bone fragments. Images were analyzed using ImageJ software.

#### Single cell RNA-sequencing library preparation

Plate based single RNA sequencing libraries were prepared using Smart-Seq2^61^ protocol with minor modification. Single BM-ECs from 2-month-old C57BL6 mice were sorted into 96-well plates containing lysis buffer (1 U/μL RNase inhibitor (Clontech), 0.1% Triton (Thermo Fisher Scientific), 2.5 mM dNTP (Invitrogen), and 2.5 μM oligo dT30VN in nuclease free water). The plates were centrifuged and frozen at −80°C. Reverse transcription was performed using SMARTScribe reverse transcriptase (Clontech) and a locked template-switching oligonucleotide (TSO), followed by 25 cycles of PCR amplification with KAPA HiFi hotStart ReadyMix (Kapa Biosystems) and ISPCR primers. The concentration and size distribution for each cell were determined by a capillary-based electrophoresis fragment analyzer (Advanced Analytical). The cDNA concentration was normalized to a range of 0.05–0.32 ng/μL using pipetting stations (Mosquito, TTP Labtech). Tagmentation and barcoding were done using Nextera XT DNA Library Kit. The libraries were pooled, purified with Agencourt AMPure XP beads, quantified using Bioanalyzer and HS-DNA Kit, and sequenced on NextSeq using single-end 1x75 reads.

10X gene expression and surface protein expression scRNA-Seq library preparation was performed using Chromium Next GEM Single Cell 3′ Reagent Kit v3.1 (10x Genomics) according to manufacturer’s instructions. BM-ECs from 2-3-month-old tamoxifen-induced C57BL6-Rainbow(R26 VT2/GK3)/Ilw x C57BL6(Cdh5-CreER)/Ilw mice (n = 6–8) were isolated and stained with live/dead dye (Live/dead Fixable Near-IR Dead Cell Stain Kit, Thermo Fisher Scientfic) according to the protocol provided by manufacturer. Next, the cells were stained with anti-CD144, anti-CD45 and anti-Ter119 antibodies as previously described, along with distinct hashing antibodies for each experimental group (TotalSeq-B, BioLegend, 0.6 μg/antibody) for 30 min on ice. Following staining 15 000 live CD45^–^Ter119^–^CD144^+^ BM-ECs were sorted per group (control, day 7, day 21 and day 60) into FACS buffer. Cells were then pooled together (control + day7, day 21+ day 60), pelleted at 400g for 5 min and loaded onto the two wells of Chromium 3′ Chip (v3.1). All samples were then processed according to Chromium Next GEM Single Cell 3′ Reagent Kits v3.1 (Dual Index) User Guide (10X Genomics) to generate cDNA sequencing libraries.

Sequencing libraries were pooled at 80% cDNA, and 20% HTO ratio. Libraries were sequenced on an Illumina NextSeq 2000 instrument using 100 cycles.

#### Bioinformatics analysis

The integrated analysis incorporated data from three distinct sources, totaling 13037 endothelial cells: 1. The bone marrow microenvironment at single-cell resolution by A. Tikhonowa et al. (GEO: GSE108892)^30^; 2. A Cellular Taxonomy of the Bone Marrow Stroma in Homeostasis and Leukemia by N. Baryawno et al. (GEO: GSE108892)^31^; 3. our Smart-seq2 scRNA-seq data. The raw Smart-seq2 sequencing data was quality checked (FastQC^62^), trimmed, and mapped with STAR^63^ default parameters to mm10 reference. Whole analysis was performed using Seurat package.^64^ Cells of low quality, defined by the detection of less than 200 genes, were excluded from each dataset. The data was then normalized using the SCT algorithm. This algorithm uses regularized negative binomial regression, which allows for the preservation of biological heterogeneity of cells while minimizing the impact of technical factors.^48^ Dimensionality reduction was then performed using principal component analysis (PCA) based on the expression of 5000 genes with the highest expression variability. The first 30 principal components were selected to create a nearest neighbor (NN) graph, which was used for clustering using the Louvain algorithm. To visualize the data, individual datasets were further reduced in dimensionality to obtain their projections in a 2D space using the UMAP algorithm based on the 30 principal components. The GSEA was done using Enrichr platform.^65^ The visualization of the data was prepared using ShinyCell package.^66^

In the study by Baryawno et al.^31^ and during our own research, sequencing of a mixture of all bone marrow cells, except for hematopoietic cells, was performed. Cells that belonged to clusters with an average expression of Cdh5 (VE-cadherin, a classical marker of endothelial cells) below 0.4 were excluded from this data.

The data was then merged using the Seurat package’s data integration method based on SCT normalization to minimize batch effects^47^^,^^67^ During the process, a common set of genes exhibiting high variability across all datasets was identified. From this set, a subset comprising 5000 genes with the most pronounced variability was selected for further analysis. Canonical correlation analysis (CCA) was then performed based on these genes to identify sources of variability present in all datasets. Subspaces were then overlaid based on the first 30 vectors of canonical correlation, and an "integrated" expression matrix was obtained, which contained the expression of the 5000 genes with the highest variability.

Based on this matrix, PCA was performed again, and a nearest neighbor graph was created based on the 30 PCA dimensions. Clustering was performed using the same method, and UMAP dimensionality reduction was performed. Since the data from the Baryawno et al.^31^ study were contained in several different files, they were also integrated and analyzed using the method described above.

The initial analysis of scRNA-seq of BM-ECs regeneration after irradiation was done with CellRanger 7.1 (10x genomics, mapped to the mm10 reference), and further processed with Seurat package.^64^ The samples were demultiplexed based on oligo Hash-tagged antibodies using HTODemux function, with modification and custom thresholds for selected Hash-tags. The data were analyzed using standard log-normal Seurat normalization and scaling, using first 25 PCA components for UMAP representation and cluster determination (with 0.5 resolution). Only cells with mitochondrial genes content below 15% and with more than 500 genes detected were included in final analysis. The few CD45^+^, erythroblasts and mesenchymal cells contaminating the endothelial pool were removed based on UMAP clustering and Cdh5 expression, before final analysis.

#### Image analysis

**Table:** 

Model	Accuracy	Sensitivity	Specificity	Precision	f1 score	Jaccard similarity	Dice coefficient
Vessels	0.847	0.819	0.834	0.816	0.758	0.666	0.758
Cells mCerulean	0.794	0.627	0.797	0.616	0.619	0.507	0.619
Cells mOrange	0.796	0.651	0.798	0.611	0.629	0.523	0.629
Cells mCherry	0.795	0.646	0.797	0.586	0.612	0.503	0.612

For image segmentation we used the Attention Residual U-Net,^68^^,^^69^^,^^70^^,^^71^ Convolutional Neural Network architecture. We used four models in total, each with 5 layers. One model was used to segment vessels, three other models were used to segment cells expressing each of the fluorescent proteins: mCerulean, mOrange and mCherry (see table above).

During training we used random rotations and reflections of the image data as augmentation. For both types of models input image patches were *Z* score normalized jointly for all channels. To increase quality of segmentation, each tile was processed 4 times with different rotations and reflections, as well as image patch blending with Hann window function – this approach was partially adapted from.^72^

Model used to segment vessels used only VE-Cadherin channel and was trained and used at half the original resolution. Base depth of the network was 32 channels.

Cell segmentation models were fed with all mCerulean, mOrange and mCherry and VE-Cadherin channels. The models were further modified to perform instance segmentation using a version of Spatial Embeddings^73^ method. It is a widely used approach^74^^,^^75^^,^^76^ where single cells are segmented by predicting offset to the cell center for each pixel within a binary cell mask. Here we achieved segmentation coordinates of cell pixels offset, iteratively refined this predicted information to aggregate them around cell centers and then assigned individual cell identities using DBSCAN clustering algorithm.^77^ We used U-Net model modifications to facilitate the segmentation. This involved addition additional residual block at maximum depth to increase receptive field of the network, and addition of two fully connected layers to achieve smoother regression results. Base depth of networks was 40 channels and each predicted binary segmentation and x,y offset to cell center.

#### Graph construction

Our goal in image processing was to extract graphs representing networks of endothelial cells, which constitute blood vessels. To do that we first divided the vessels into domains corresponding to individual cells. Because fluorescent proteins in our images were mostly concentrated in nuclei, these domains were also a more accurate representation of individual cells.

We calculated a map of geodesic distance from cells’ boundaries within the vessel mask, using fast marching algorithm,^78^ creating low value basins around segmented instances. By running a watershed^79^ algorithm on this distance map, with segmented cells as markers, we were able to obtain the aforementioned domains (Figure S3F).

We then constructed region adjacency graphs^79^ (Figure S3G) based on those domains for each image. Those graphs were saved for later analysis, along with measured geodesic distances between adjacent cells, and colors and locations of each cell.

#### Predicting regeneration parameters

##### Local assortativity measurement

The key to decode the information about BM-ECs regeneration, which is contained within cell graphs, was the evaluation of cells’ preference for contacts with cells of the same color. The preferentiality of those contacts can be calculated as global assortativity of a given labeled graph.^80^ Global assortativity (r_global_) formula for unweighted graph is:

$$r_{global}=\frac{\sum_{g}e_{gg}-\left\|a^{2}\right\|}{1-\left\|a^{2}\right\|}$$

Where $e_{gh}$ is a fraction of edges connecting nodes with label g to nodes with label h, and a is a matrix:

$$a=\left[\begin{array}{ccc} e_{11} & \cdots & e_{1n}\\ \vdots & \ddots & \vdots \\ e_{n1} & \cdots & e_{nn} \end{array}\right]$$

where n is a number unique of label. This metric was proved to be sufficient for very simple approximation of regenerative process; however, it couldn’t reflect more realistic simulations. As such we used local assortativity, which instead of describing assortative mixing in the whole graph, focuses on a neighborhood of a given node l.^81^ For this evaluation we assigned weights to edges that reflect their locality to the node l. The modified variable $e_{gh}$ is subsequently introduced, which considers locality of a given edge to the node ( ω(i;l) ):

$$e_{gh}\left(l\right)=\sum_{i:y_{i}=g}\sum_{j:y_{j}=h}\omega \left(i;l\right)\frac{A_{ij}}{\deg\;\left(i\right)}$$

Thus, local assortativity of the node l is defined as:

$$r_{local}\left(l\right)=\frac{\sum_{g}e_{gg}\left(l\right)-\left\|a^{2}\right\|}{1-\left\|a^{2}\right\|}$$

As evidenced by the formula for $e_{gh}\left(l\right)$ the weight of an edge depends on the weight of the node it originates form (actual computation for undirected graph, treats each undirected edge as two directed edges in opposite directions). The weight of a node (i.e., its locality to node l; ω(i;l)) is calculated using personalised page rank algorithm,^82^ with the point of return being node l. Damping factor of pagerank algorithm controles range of locality, with low values limiting it only to immediate neighbors, and for values approaching 1 extending it to whole graph.

Through this calculation we obtained values of $r_{local}$ for each node in a given graph. By examining distribution of $r_{local}$, we could more accurately describe preferential connections within a graph.

#### Regeneration simulation

To create the model capable of predicting original graphs’ regeneration we first established a dataset of ground truth (GT) values. As the structure of a graph impacts local assortativity values, we generated individual GT dataset for each graph, by simulating clonal expansion on existing network, in accordance with the regeneration parameters, while changing only color labels of the node. Our parameters were progenitor fraction – the fraction of initial cells that started proliferation, and new cells fraction – the fraction of new cells in observed network, which derive from the cells that started proliferation (actual prediction was 1-new cells fraction).

In our simulation we assumed that daughter can only be present in the immediate neighborhood of their mother cell. Given that endothelial cells create continuous monolayer within the vessels, this simulation more accurately reflects the biological context compared to a simulation where new cells can move to fill spaces distant from their mother cell. We also compared both types of simulations, and the first produced graph colorings far more similar to our experimental dataset.

During the simulation, we first assigned each graph node a unique identity and randomly assigned them roles of either new cells, progenitor cells, or passive cells (Figure S3H). We then calculated the optimal rearrangement of initial labels by moving new cells closer to progenitor cells. This was done by calculating a gradient originating from progenitor cells using personalized page rank, and iteratively swapping labels between passive cells and new cells, "pushing" passive cells away. This process continued until there were no edges where the gradient pointed from a passive cell to a new cell, and all new cells were close to progenitors (Figure S3I).

Next, the graph was divided into unique domains to be reconstructed by each progenitor cell. The personalized page rank of each progenitor cell node was calculated and nodes where each progenitor had the highest page rank value became their domain (Figure S3J, already assigned colors). New cell nodes were then stripped of their original identities and given the identity of the domain they fell within. Each cell was randomly assigned a color based on its new identity, with new daughter cells gaining the color of their progenitor cell (Figure S3K).

#### Predicting parameters

We predicted regeneration characteristics for each graph with more than 70 nodes, which corresponded to sufficiently large unconnected vessel fragments in the image. For the model data, we utilized moments of the local assortativity distribution (mean, variance, skewness and kurtosis), several quantiles and correlation between distributions of local assortativities computed using pagerank with different damping factors. We used damping factors, 0.1 and 0.7, however we found, that our approach was largely insensitive to the damping factor selection in the local assortativity equation. Parameters of local assortativity distributions were *Z* score normalized and PCA was performed to reduce dimensionality of data (Figure S3N).

Because of the significant impact of graph’s shape and size on assortativity distributions, we used unique models for each graph, instead of a general model. Our model of choice was a feedforward neural network (FNN) and we trained it to output predictions as Gaussian distributions instead of single points with the mean-variance estimation (MVE) method.^83^ This method is based on maximum-likelihood estimation and the model was trained with negative log likelihood loss to output both predictions and their log variances. This allowed us to estimate confidence level of a given prediction based on width of a Gaussian distribution.

To keep the entirety of predicted distributions within the range 0–1 and avoid illogical predictions, as well as to improve accuracy of predictions, FNN was predicting logits of target values. This resulted in predicted uncertainty distributions for target values being logit-normal distributions, which was considered in the downstream analysis.

To further confirm validity of predictions for original graphs we calculated Bhattacharyya distance between empirical distributions of their assortativities and those of simulated graphs. It allowed us to confirm that the model predictions fell within the most probable values.

### Quantification and statistical analysis

Flow cytometry data were analyzed using FlowJo, Microsoft Excel (Microsoft) and GraphPad Prism 8 software.

LDA data were analyzed with ELDA algorithm available at https://bioinf.wehi.edu.au/software/elda/ and as an R package.^49^

RNA sequencing data as well as ECs’ regeneration analyses are described in the single-cell RNA sequencing and Image analysis sections. Details concerning statistical tests can be found in figures’ legends. The data are presented as mean ± SEM, unless otherwise stated. All experiments were repeated as indicated; n indicates the number of independent biological repeats, unless otherwise stated. Randomization and blinding were used in case of quantitative and qualitative analysis of *in vitro* vessel-like structure formation. No statistical method was used to predetermine sample size. To test statistical significance between two groups, a two-tailed Student’s t-test was used, unless otherwise stated. For enrichment analysis in Figure 3E Hypergeometric test with Bonferroni correction for multiple comparisons was performed. The reported *p*-values in Figures 3F and 3G, S3B–S3G, are obtained using Enrichr tool^65^^,^^84^ that applies Fisher exact test with assumed binominal distribution adjsuted by Benjamini-Hochberg method for multiply testing.

## Supplemental information

Document S1. Figures S1–S5

Document S2. Article plus supplemental information
