# Polyclonal regeneration of mouse bone marrow endothelial cells after irradiative conditioning

**DOI:** 10.1016/j.celrep.2026.117448

**Published:** 2026-05-30

**Authors:** Izabella Skulimowska, Jan Morys, Justyna Sosniak, Monika Gonka, Gunsagar Gulati, Rahul Sinha, Kacper Kowalski, Sylwester Mosiolek, Irving L. Weissman, Alicja Jozkowicz, Agata Szade, Krzysztof Szade

## Main text

(Cell Reports *43*, 114779; November 26, 2024)

There is an error in the “bioinformatics analysis” section of the method details subsection of the STAR Methods in this paper. The datasets from Tikhonova et al. and Baryawno et al. are both given a GEO number of GSE108892, but Barywano et al.’s GEO deposition should be listed as GSE128423.

Although the paper cannot be updated at this time due to the amount of time that has passed since its initial publication, the authors agreed to publish a correction notice to point readers to the correct deposition.

