# Supplementary material for: Polyclonal regeneration of mouse bone marrow endothelial cells after irradiative conditioning
Source: Cell Rep. 2024 Oct 26;43(11):114779. doi: 10.1016/j.celrep.2024.114779 (PMC11602546; doi:10.1016/j.celrep.2024.114779)
Supplement: Document S1. Figures S1–S5 [file mmc1.pdf]

Cell Reports, Volume *43*

Supplemental information

Polyclonal regeneration of mouse bone marrow endothelial cells after irradiative conditioning

Izabella Skulimowska, Jan Morys, Justyna Sosniak, Monika Gonka, Gunsagar Gulati, Rahul Sinha, Kacper Kowalski, Sylwester Mosiolek, Irving L. Weissman, Alicja Jozkowicz, Agata Szade, and Krzysztof Szade

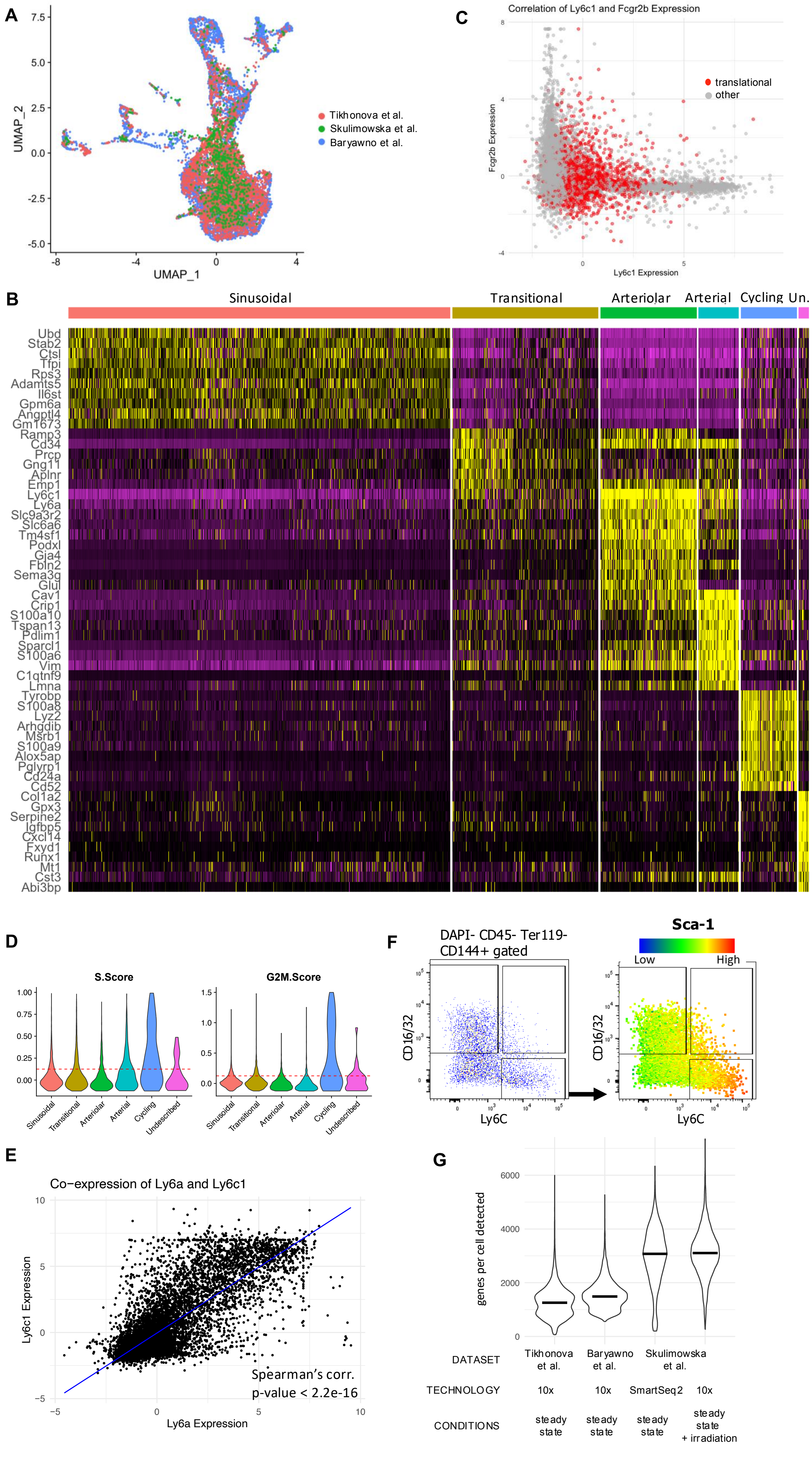
A
UMAP_2
UMAP_1
Tikhonova et al.
Skulimowska et al.
Baryawno et al.
C
Correlation of Ly6c1 and Fcgr2b Expression
Fcgr2b Expression
Ly6c1 Expression
translational
other
B
Sinusoidal
Transitional
Arteriolar
Arterial
Cycling
Un.
Ubd
Stab2
Ctsl
Tfpi
Rps3
Adamts5
Il6st
Gpm6a
Angptl4
Gm1673
Ramp3
Cd34
Prcp
Gng11
Aplnr
Emp1
Ly6c1
Ly6a
Slc9a3r2
Slc6a6
Tm4sf1
Podxl
Gja4
Fbln2
Sema3g
Glul
Cav1
Crip1
S100a10
Tspan13
Pdlim1
Sparcl1
S100a6
Vim
C1qtnf9
Lmna
Tyrobp
S100a8
Lyz2
Arhgdib
Msrb1
S100a9
Alox5ap
Pglyrp1
Cd24a
Cd52
Col1a2
Gpx3
Serpine2
Igfbp5
Cxcl14
Fxyd1
Runx1
Mt1
Cst3
Abi3bp
D
S.Score
G2M.Score
Sinusoidal
Transitional
Arteriolar
Arterial
Cycling
Undescribed
F
DAPI- CD45- Ter119- CD144+ gated
Sca-1
Low
High
CD16/32
Ly6C
E
Co−expression of Ly6a and Ly6c1
Ly6c1 Expression
Ly6a Expression
Spearman's corr.
p-value < 2.2e-16
G
genes per cell detected
DATASET
Tikhonova et al.
Baryawno et al.
Skulimowska et al.
TECHNOLOGY
10x
10x
SmartSeq2
10x
CONDITIONS
steady state
steady state
steady state
steady state + irradiation

Supplementary Figure 1

Analysis of integrated sc-RNAseq data including mouse bone marrow endothelial cells under steady-state conditions.

(A) UMAP projection showing the distribution of cells derived from different datasets. **(B)** Top differentially expressed genes for each cluster, identified with ROC analysis. UN. – undesrcibed **(C)** Expression of Fcgr2b and Ly6c1 in all cells. **(D)** S and G2/M scores for each cluster **(E)** Correlation of Ly6c1 and Ly6a in integrated scRNA-seq datasets **(F)** Correlation of CD16/32 and Sca-1 expression in BM-ECs evaluated by flow cytometry analysis. **(G)** Number of detected genes among datasets used and generated in the study.

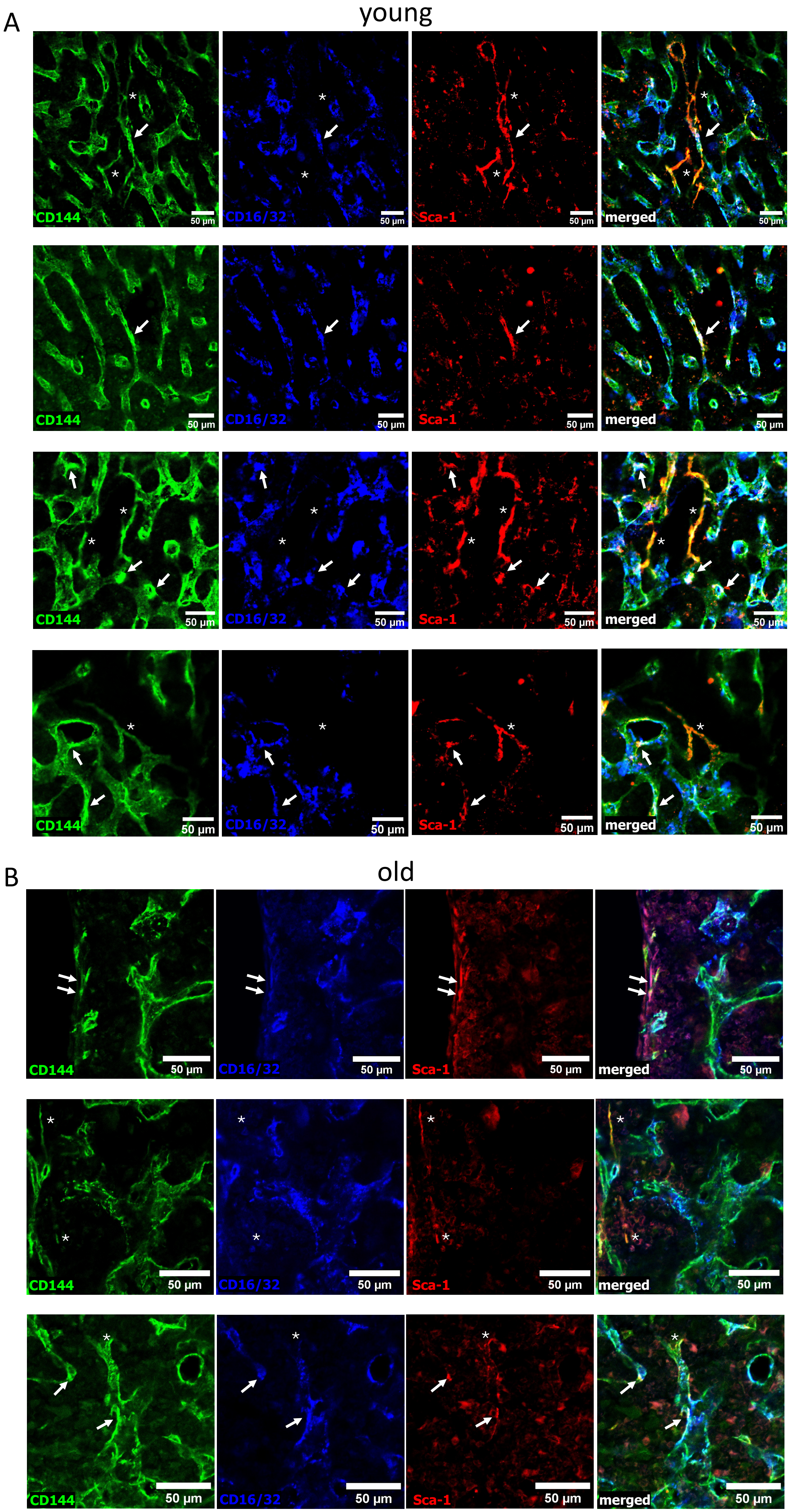
A
young
CD144
CD16/32
Sca-1
merged
50 μm
B
old
CD144
CD16/32
Sca-1
merged
50 μm

Supplementary Figure 2

Examples of transitional Sca-1/Ly6A^{high} CD16/32^{+} BM-ECs detected by immunohistochemistry. (A) Young mice. **(B)** Old mice. The transitional BM-ECs localized between arterioles Sca-1/Ly6A^{high} CD16/32^{-} (*) that along the vessel gradually change into transitional double positive Sca-1/Ly6A^{+} CD16/32^{+} BM-ECs (arrow), before becoming wider Sca-1/Ly6A$^{-/low}$ CD16/32^{+} sinusoids.

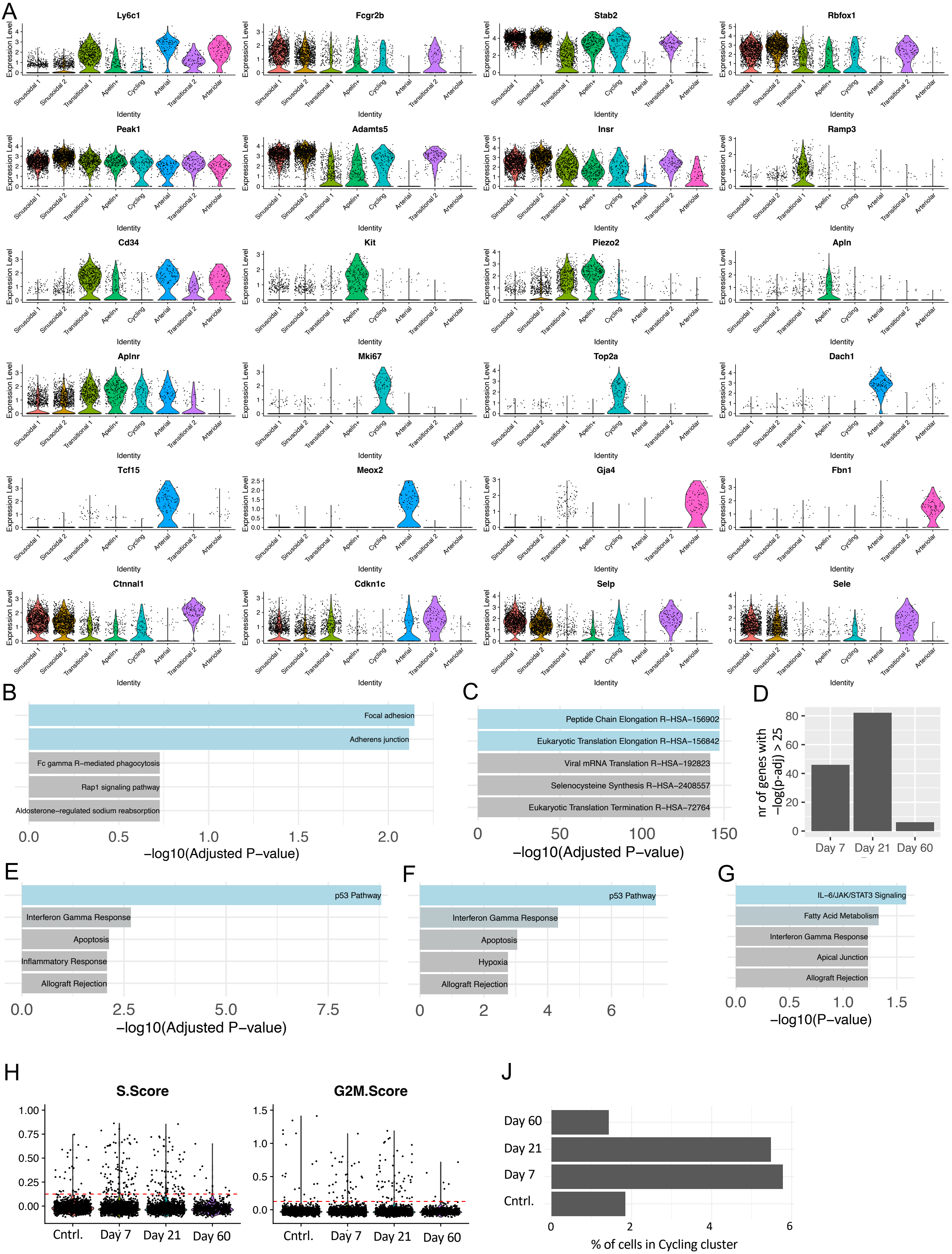
A
Ly6c1
Fcgr2b
Stab2
Rbfox1
Peak1
Adamts5
Insr
Ramp3
Cd34
Kit
Piezo2
Apln
Aplnr
Mki67
Top2a
Dach1
Tcf15
Meox2
Gja4
Fbn1
Ctnnal1
Cdkn1c
Selp
Sele
Expression Level
Sinusoidal 1
Sinusoidal 2
Transitional 1
Apelin+
Cycling
Arterial
Transitional 2
Arteriolar
Identity
B
Focal adhesion
Adherens junction
Fc gamma R−mediated phagocytosis
Rap1 signaling pathway
Aldosterone−regulated sodium reabsorption
0.0
0.5
1.0
1.5
2.0
−log10(Adjusted P−value)
C
Peptide Chain Elongation R−HSA−156902
Eukaryotic Translation Elongation R−HSA−156842
Viral mRNA Translation R−HSA−192823
Selenocysteine Synthesis R−HSA−2408557
Eukaryotic Translation Termination R−HSA−72764
0
50
100
150
−log10(Adjusted P−value)
D
nr of genes with −log(p-adj) > 25
0
20
40
60
80
Day 7
Day 21
Day 60
E
p53 Pathway
Interferon Gamma Response
Apoptosis
Inflammatory Response
Allograft Rejection
0.0
2.5
5.0
7.5
−log10(Adjusted P−value)
F
p53 Pathway
Interferon Gamma Response
Apoptosis
Hypoxia
Allograft Rejection
0
2
4
6
G
IL−6/JAK/STAT3 Signaling
Fatty Acid Metabolism
Interferon Gamma Response
Apical Junction
Allograft Rejection
0.0
0.5
1.0
1.5
−log10(P−value)
H
S.Score
G2M.Score
Cntrl.
Day 7
Day 21
Day 60
J
Day 60
Day 21
Day 7
Cntrl.
0
2
4
6
% of cells in Cycling cluster

Supplementary Figure 3

Transcriptional changes in bone marrow endothelial cells during regeneration revealed by sc-RNAseq data analysis.

(A) Expression of selected cluster markers among the identified clusters **(B)** GSEA analysis on markers genes of Sinusoidal cluster using KEGG 2021database. **(C)** GSEA analysis on differentially expressed genes between Sinusoidal 2 and Sinusoidal 1 clusters using Reactome database. **(D)** Number of highly differentially expressed genes (-log(p-adj)>25) in different time points post-irradiation when compared to control cells. **(E-G)** GSEA analysis on differentially expressed genes at day 7 **(E)**, day 21 **(F)**, and day 60 **(G)** post-irradiation (MSigDB). **(H)** S and G2/M scores for different experimental groups and set threshold (red dashed line) for classification as cycling cells. **(J)** Frequency of cells in the Cycling cluster in different experimental groups.

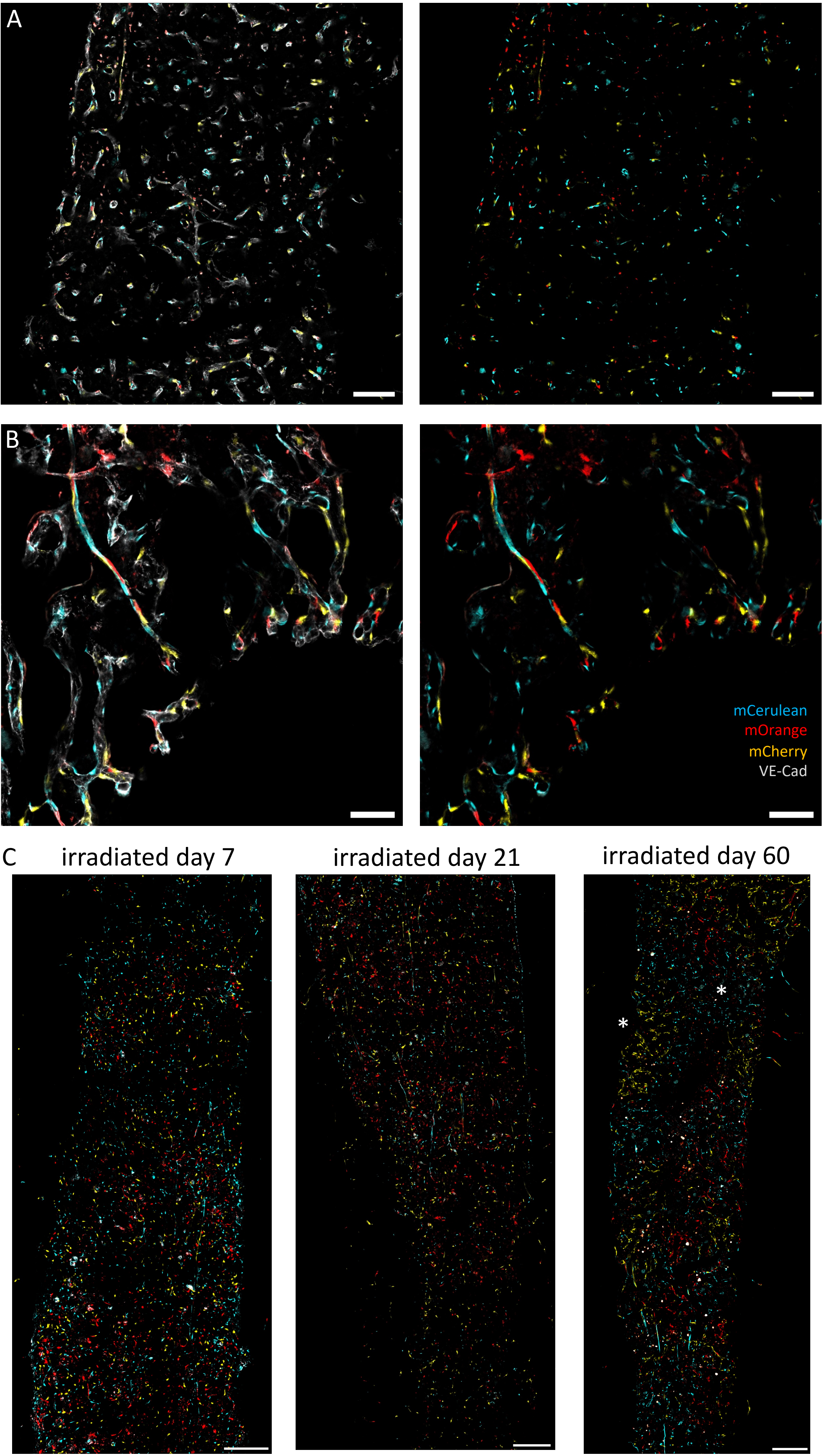

A
B
mCerulean
mOrange
mCherry
VE-Cad
C
irradiated day 7
irradiated day 21
irradiated day 60
*
*

Supplementary Figure 4

Expression pattern of fluorescent proteins in non-irradiated and irradiated BM.

White – VE-Cadherin, blue – mCerulean, orange – mOrange, red – mCherry.

(A,B) The expression of random fluorescent markers upon induction was specific to ECs and labelled uniquely ECs in **(A)** diaphysis and **(B)** metaphysis region of the bone. Bars 100 μm and 50 μm respectively.

(C) We did not observe any regions with one color at 7 or 21 days after irradiation, however there were areas with single color visible 60 days post-irradiation (*). Bars 200 μm.

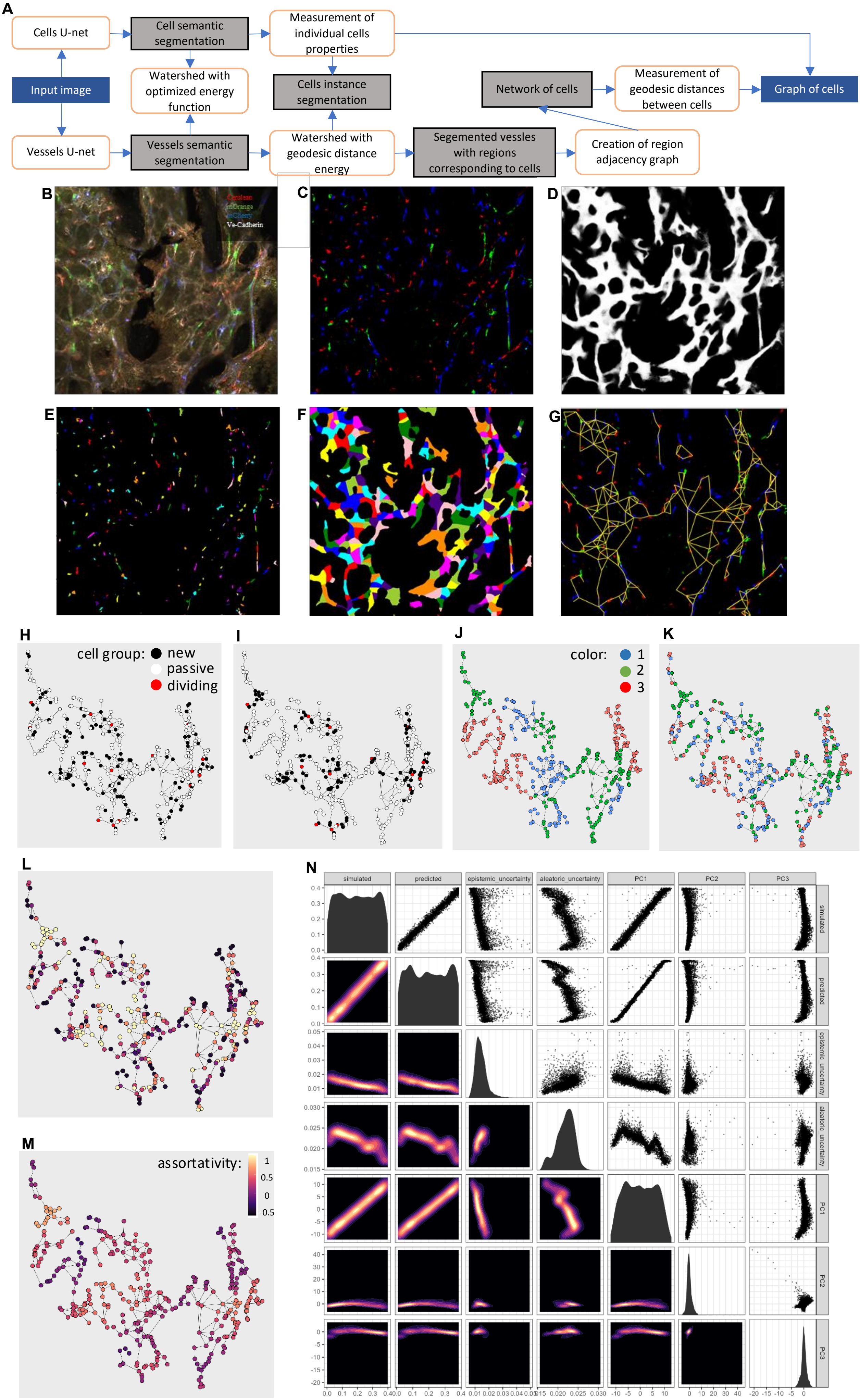

A
Cells U-net
Cell semantic segmentation
Measurement of individual cells properties
Input image
Watershed with optimized energy function
Cells instance segmentation
Network of cells
Measurement of geodesic distances between cells
Graph of cells
Vessels U-net
Vessels semantic segmentation
Watershed with geodesic distance energy
Segemented vessles with regions corresponding to cells
Creation of region adjacency graph
B
Cerulean
mOrange
mCherry
Ve-Cadherin
C
D
E
F
G
H
cell group: new passive dividing
I
J
color: 1 2 3
K
L
M
assortativity: 1 0.5 0 -0.5
N
simulated
predicted
epistemic_uncertainty
aleatoric_uncertainty
PC1
PC2
PC3

Supplementary Figure 5

Strategy applied for image segmentation, graph constructions, and model training.

(A) Overview of the proposed pipeline. U-Net for cells segmentation uses as an input an entire, 4 channel, image **(B)**, while U-Net for vessel segmentation uses only Ve-Cad channel. Cell semantic segmentation **(C)** and vessel segmentation **(D)** images are then used to perform instance segmentation of cells **(E)**. Cell instance segmentation and vessels segmentation are used in watershed transformation, with the watershed energy being geodesic distance from segmented cells. Through this transformation we obtain map of segmented vessels with regions corresponding to cells **(F)**, which we use to create region adjacency graph **(G)**. Region adjacency graph is then used to identify neighbours of each cell and measure distance between to them. Finally, all data about cells and the neighbourhood is pooled to create a graph.

(H-M) Simulation of vessel regeneration.

(H) The first step of the simulation randomly chooses surviving and dividing (progenitor) cells among them, here respectively 30% and 6%.

(I) Then the iterative "push" algorithm rearranges positions of passive cells, to create patches of new cells around progenitors, mimicking patches observed in experimental data.

(J) Domains with empty nodes covered by daughter cells from given proliferating cells were defined through page rank algorithm (colors for clarity).

(K) Random colors are then assigned to cells based on the identifier, completing the simulation.

(L,M) Local assortativity can be used to examine preferential contacts of cell with other cells of the same colors, indicating clonal expansion. **(L)** Low values of damping factor in the page rank algorithm (implemented in the local assortativity equation) tune the algorithm to examine if the color of a given cell is the same as the color of cells in close neighborhood, **(M)** while larger values

of the damping factor tune the algorithm to examine if the color of a given cell is the same as the color of cells within broader neighborhood area including more distant cells. Thus use of large dumping factor results in smoothing the local assortativity values over the graph.

(N) Predicting parameters. Data describing assortativity distribution for a given graph labelling is already well correlated with input parameters, especially for new cell fraction prediction, however model can still improve the prediction, especially for the outliers. Majority of the prediction uncertainty is aleatoric stemming from randomness in color selection, and increases with survival rate.
